# Reprogramming NK cells and macrophages via combined antibody and cytokine therapy primes tumors for elimination by checkpoint blockade

**DOI:** 10.1016/j.celrep.2021.110021

**Published:** 2021-11-23

**Authors:** Chensu Wang, Ang Cui, Maurice Bukenya, Aereas Aung, Dikshant Pradhan, Charles A. Whittaker, Yash Agarwal, Ayush Thomas, Simon Liang, Parastoo Amlashi, Heikyung Suh, Stefani Spranger, Nir Hacohen, Darrell J. Irvine

**Affiliations:** 1 Koch Institute for Integrative Cancer Research, Massachusetts Institute of Technology, Cambridge, MA, USA; 2 Broad Institute of MIT and Harvard, Cambridge, MA, USA; 3 Harvard-MIT Division of Health Sciences and Technology, MIT, Cambridge, MA, USA; 4 Department of Biological Engineering, Massachusetts Institute of Technology, Cambridge, MA, USA; 5 Department of Biology, Massachusetts Institute of Technology, Cambridge, MA, USA; 6 Center for Cancer Research, Massachusetts General Hospital and Harvard Medical School, Boston, MA, USA; 7 Ragon Institute of Massachusetts General Hospital, Massachusetts Institute of Technology and Harvard, Cambridge, MA, USA; 8 Department of Materials Science and Engineering, Massachusetts Institute of Technology, Cambridge, MA, USA; 9 Howard Hughes Medical Institute, Chevy Chase, MD, USA; 10 Lead contact

## Abstract

Treatments aiming to augment immune checkpoint blockade (ICB) in cancer often focus on T cell immunity, but innate immune cells may have important roles to play. Here, we demonstrate a single-dose combination treatment (termed AIP) using a pan-tumor-targeting antibody surrogate, half-life-extended interleukin-2 (IL-2), and anti-programmed cell death 1 (PD-1), which primes tumors to respond to subsequent ICB and promotes rejection of large established tumors in mice. Natural killer (NK) cells and macrophages activated by AIP treatment underwent transcriptional reprogramming; rapidly killed cancer cells; governed the recruitment of cross-presenting dendritic cells (DCs) and other leukocytes; and induced normalization of the tumor vasculature, facilitating further immune infiltration. Thus, innate cell-activating therapies can initiate critical steps leading to a self-sustaining cycle of T cell priming driven by ICB.

## In brief

Wang et al. report an immune priming therapy based on a single dose of anti-tumor antibodies, IL-2, and anti-PD-1, which engages natural killer cells and macrophages, promotes lymphocyte recruitment and activation, and elicits vascular normalization. This priming strategy allows subsequent immune checkpoint blockade (ICB) to eradicate large, established tumors.

## Graphical Abstract

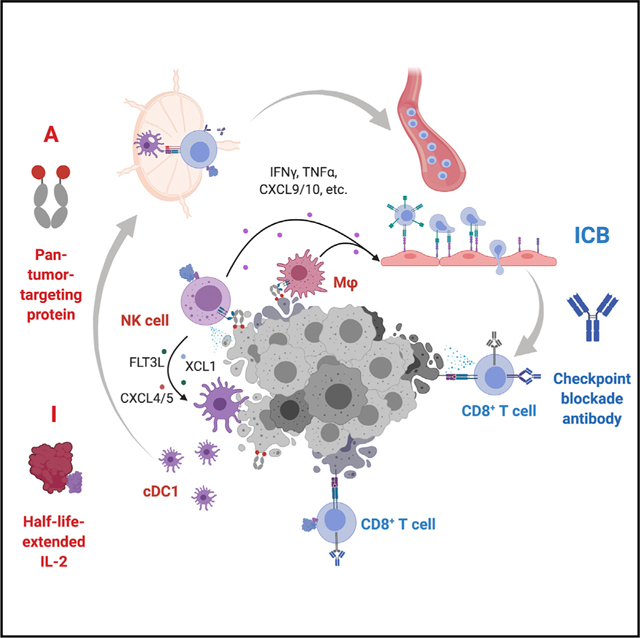

## INTRODUCTION

Immune checkpoint blockade (ICB) therapies based on the administration of antibodies against the T cell inhibitory molecules cytotoxic T lymphocyte-associated protein 4 (CTLA-4) and programmed cell death 1 (PD-1, or its ligands) have been approved for the treatment of a variety of malignancies alone or in combination (Antonia et al., 2017; Hellmann et al., 2018; Hodi et al., 2010; Reck et al., 2016; Socinski et al., 2018). However, in most tumors, ICB benefits a minority of patients, and only a subset of responders exhibit durable responses (Gao et al., 2016; George et al., 2017; Sade-Feldman et al., 2017; Zaretsky et al., 2016). To better understand the underlying mechanisms driving these variations in response, substantial work has been carried out to identify features of the tumor microenvironment (TME) that correlate with long-term survival and predict responses to ICB (Le et al., 2015; Sade-Feldman et al., 2018; Snyder et al., 2014; Tumeh et al., 2014; Yuen et al., 2020). One factor associated with outcome is the number of infiltrating T cells prior to or during early treatment (Chen et al., 2016; Tumeh et al., 2014), introducing the notion of “hot” (inflamed and highly infiltrated) and “cold” (immunosuppressive and non-infiltrated) tumors (Galon et al., 2006). Those patients responding to ICB most often exhibit a “hot” tumor phenotype.

These findings have led to an intensive search for combination treatments that could convert “cold” tumors into the “hot” lymphocyte-infiltrated state, under the assumption that such co-treatments would increase the proportion of ICB-responding patients. To date, a variety of immunotherapeutic strategies have been studied that provide some level of benefit when combined with ICB treatment in preclinical models or small clinical studies (Carmi et al., 2015; Ribas et al., 2017), but in general the synergies discovered have been modest and the immunological ruleset to promote ICB responsiveness remains poorly defined. Recently, we identified a potent four-component immunotherapy comprised of an anti-tumor antibody (“A”), half-life-extended interleukin-2 (“I”), anti-PD-1 (“P”), and a peptide vaccine (“V,” with the combination treatment abbreviated as AIPV hereafter), which cured a majority of animals in several difficult-to-treat transplanted and genetically engineered mouse models (GEMMs) of cancer (Moynihan et al., 2016). Mechanistically, this treatment was dependent not only on CD8^+^ T cells but also several subsets of innate immune cells, including macrophages and natural killer (NK) cells, suggesting the importance of effectively engaging adaptive and innate immune responses against tumors in tandem. However, clinical translation of this therapy is complicated by its multicomponent nature and the need for multiple tumor-specific therapeutic components (i.e., the “A” and “V”).

Motivated by the striking changes induced in the TME following a single dose of AIPV therapy, we hypothesized that a treatment simultaneously activating innate and adaptive responses might prime tumors to respond to treatment with ICB alone. Here, we tested AIPV and its subcombinations as a single-dose combination treatment prior to ICB and discovered that a simplified regimen combining a pan-tumor integrin-targeting antibody surrogate molecule with long half-life IL-2 and anti-PD-1, administered once, triggered remodeling of the TME, induction of rapid tumor cell death, and initiated T cell priming in tumor-draining lymph nodes. Mechanistically, these changes were mediated primarily by NK cells and macrophages, which induced rapid tumor antigen release to dendritic cells (DCs), mediated immune infiltration, and promoted vascular normalization. These findings suggest that a relatively simple combination treatment may be capable of substantially increasing responsiveness to checkpoint blockade, by exploiting coordinated functions of innate immune cells together with T cell functionality in the TME.

## RESULTS

### A single dose of AIP immunotherapy inflames the TME and sensitizes tumors to checkpoint blockade

**Figure 1.**
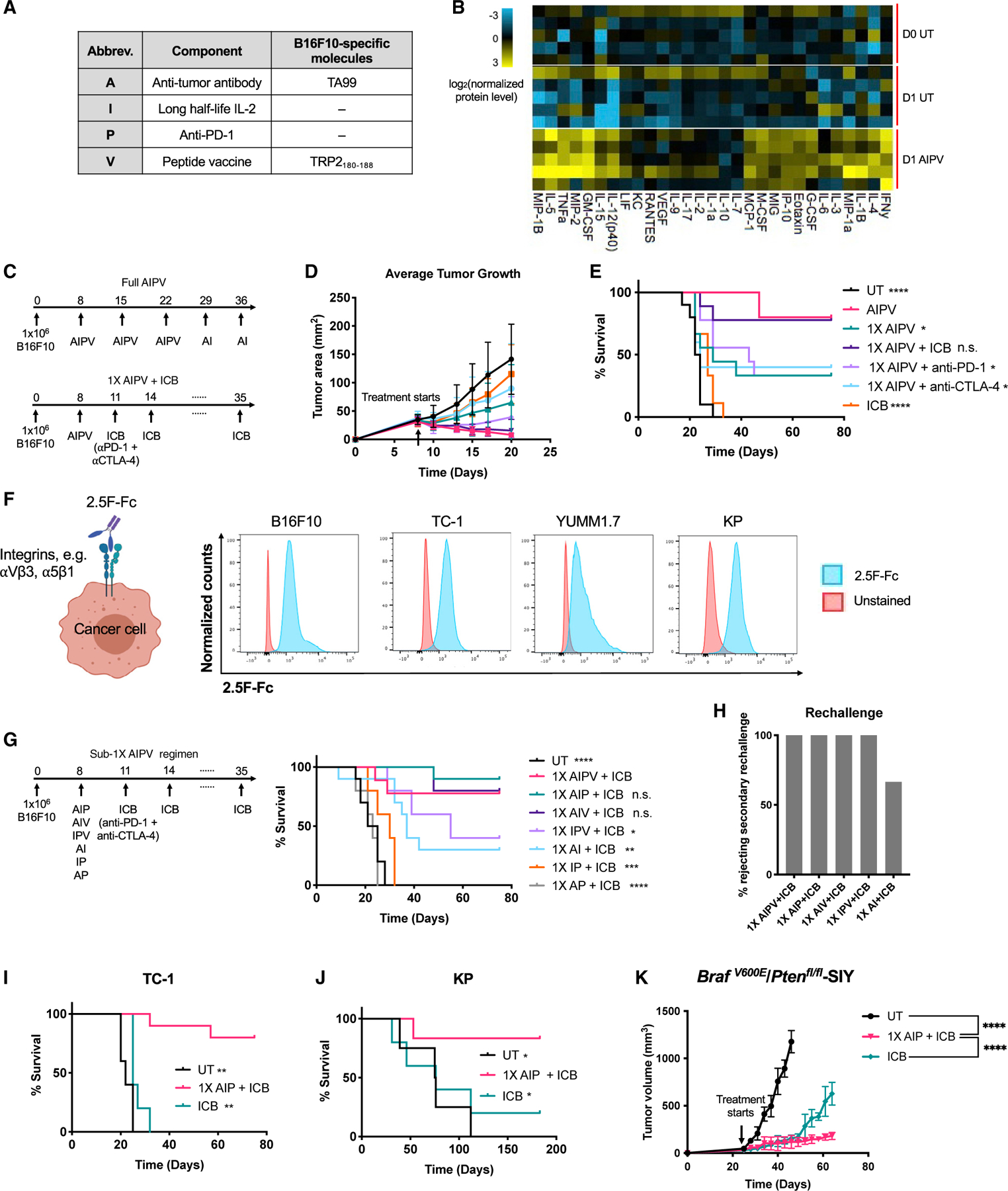
Single-dose AIP immunotherapy sensitizes tumors to subsequent immune checkpoint blockade (ICB) (A) Components of AIPV therapy. (B) C57BL/6J mice (n = 5/group) were inoculated with 1 × 10^6^ B16F10 cells in the flank. Eight days later when tumors were 40–50 mm^2^ (D0), animals were treated with AIPV therapy or left untreated (UT); tumors were analyzed on D0 and 1 day later for cytokine/chemokine levels by Luminex. Shown is log_2_ (normalized protein level/column mean). (C–E) C57BL/6J mice with established B16F10 tumors as in (B) were treated with AIPV or 1X AIPV followed by checkpoint blockade administered every 3 days. Shown are timelines of AIPV and 1X AIPV regimens (C), average tumor size (D), and survival (E) over time. (F) Tumor cells were stained with AF647-labeled 2.5F-Fc and analyzed by flow cytometry. (G and H) C57BL/6J mice (n = 5–10 animals/group) bearing B16F10 tumors as in (B) were treated with subcombinations of 1X AIPV therapy using 2.5F-Fc as the “A” component, followed by ICB. Shown are timelines of 1X sub-AIPV regimens and survival over time (G) and percentages of long-term survivors who rejected a rechallenge with 10^5^ B16F10 tumor cells on day 75 (H). (I and J) Survival over time for C57BL/6J mice inoculated with 10^6^ TC-1 (I) cells subcutaneously (s.c.) in the flank or 0.5 × 10^6^ KP (*Kras*^LSL-G12D/+^;*p53*^fl/fl^) tumor cells implanted intratracheally into the lung (J) and treated 8 days later with AIP followed by ICB or ICB alone. (K) Melanoma in *Braf*^*V600E*^/*Pten*^*fl/fl*^-SIY (BP-SIY) mice (n = 5 animals/group) was initiated by application of 4-hydroxytamoxifen on day 0. Shown is the average tumor volume of the UT mice or mice treated with 1X AIP or ICB starting on day 25, following the same schedule as in (G). In (C) to (J), n = 5–10 animals/group pooled from two independent experiments unless otherwise indicated. *p < 0.05; **p < 0.01; ***p < 0.001; ****p < 0.0001 versus AIPV or single-dose AIP by log rank test unless otherwise indicated. All error bars are standard deviation (SD). See also Figures S1 and S2.

We previously reported that weekly-administered AIPV therapy induced dramatic changes in the TME, leading to eradication of large established tumors in a range of syngeneic cancer models (Moynihan et al., 2016). To obtain greater insight into the kinetics of changes to the TME following treatment with this combination therapy, we carried out Luminex-based analysis of cytokine and chemokine levels in tumors at early time points after AIPV administration in the B16F10 melanoma model. We used the anti-TYRP1 antibody TA99 as the “A” component and a peptide vaccine against the TRP2_180–188_ peptide as the “V” component (Figure 1A). Strikingly, we found that a broad range of pro-inflammatory cytokines were upregulated within 1 day of treatment, including interferon γ (IFN-γ), tumor necrosis factor alpha (TNF-α), and interleukin-12 (IL-12) as well as key chemokines including CCL3/4, CCL5, CXCL9, and CXCL10 (Figure 1B; Figure S1A). CD4^+^ and CD8^+^ T cells, NK cells, conventional type 1 dendritic cells (cDC1s) and cDC2s, NK cells, and neutrophils were all recruited to the tumor over the course of 6 days following a single dose of AIPV, and the CD8/Treg ratio shifted dramatically (Figures S1B and S1C). We previously studied AIPV administered as a multi-dose regimen over 5 weeks (“full AIPV” regimen; Figure 1C). Encouraged by the ability of a single dose of AIPV to inflame relatively cold B16F10 tumors within a few days, we evaluated whether a single dose of AIPV followed by clinically approved ICB with anti-PD-1 and anti-CTLA-4 could be effective (“1X AIPV + ICB”; Figure 1C). Strikingly, single-dose AIPV administered to animals bearing large ~40–50 mm^2^ tumors followed by PD-1 and CTLA-4 blockade (hereafter, ICB) led to tumor regressions comparable with the “full” AIPV regimen, with complete responses in 80% of animals (Figures 1D and 1E). ICB alone had no impact on overall survival in this setting, and 1X AIPV without subsequent ICB dosing or treatments applying 1X AIPV followed by anti-PD-1 alone or anti-CTLA-4 alone were much less effective than AIPV followed by combined checkpoint blockade (Figures 1D and 1E). The full AIPV regimen elicited high levels of TRP2-specific T cells (the antigen targeted by the “V” component) after repeated dosing, and induced antigen spreading to epitopes expressed by B16F10 cells other than TRP2 (Figures S1D and S1E). 1X AIPV therapy elicited much weaker TRP2-directed T cell responses, but elicited equivalent levels of T cell responses against other B16F10 epitopes (Figures S1D and S1E). Hence, a single dose of the AIPV combination was sufficient to render intractable B16F10 tumors susceptible to ICB.

We next sought to determine whether this combination 1X AIPV “priming” treatment could be made simpler and more amenable for pan-tumor treatment. We first replaced the melanoma-specific TA99 antibody with a recently described pan-tumor integrin-binding protein 2.5F-Fc, an engineered integrin-targeting cysteine knot peptide fused to a mouse IgG2c Fc domain (Van Agthoven et al., 2019; Kimura et al., 2009; Kwan et al., 2017; Moore et al., 2013) (Figure 1F). This antibody surrogate recognizes α_5_β_1_ and multiple α_v_-containing integrins that are overexpressed in many human and mouse tumors, and it stained B16F10 melanoma, TC-1 HPV E6/E7-expressing tumor cells (Lin et al., 1996), the YUMM1.7 melanoma cell derived from the *Braf*^*V600E*^*/Pten*^*fl/fl*^ GEMM (Meeth et al., 2016), and KP lung cancer cells generated from the *LSL-Kras*^*G12D/+*^*/p53*^*fl/fl*^ GEMM (Figure 1F). We tested 1X AIPV using this revised “A” component as well as each of the triple- and two-component subcombinations of the regimen for their ability to prime B16F10 tumors for eradication by ICB. These experiments revealed that one dose of AIP or AIV prior to ICB was as effective as AIPV for curing large B16 tumors, while IPV or two-component treatments were substantially less effective (Figure 1G). Animals cured by 1X AIPV, AIP, AIV, or IPV followed by ICB had protective memory and rejected tumor rechallenge (Figure 1H). As the “V” remains a tumor-specific treatment component, we opted to focus on 1X AIP as a combination for further study. In the B16F10 model, the 1X AIP + ICB combination therapy generated substantially stronger tumor-specific T cell responses than ICB alone or in mice left untreated by 1-week post dosing (Figure S1F). A broad array of inflammatory cytokines and chemokines were induced one day following AIP treatment using the 2.5F-Fc antibody surrogate, comparable with AIPV therapy (Figure S2A). 1X AIP followed by ICB also elicited high rates (80%–100%) of complete cures of large established TC-1 and YUMM1.7 flank tumors and orthotopic KP lung tumors, where ICB alone was either only partially effective (KP) or completely ineffective (TC-1 and YUMM1.7) (Figures 1I and 1J; Figure S2B). We further assessed the efficacy of the 1X AIP treatment in an autochthonous *Braf*^*V600E*^/*Pten*^*fl/fl*^ melanoma model that better mimics the etiology and progression of human melanoma. In this model, tamoxifen applied to the skin of mice induces expression of Cre in melanocytes, which in turn activates expression of mutant Braf, Pten deletion, and expression of a defined H-2K^b^-restricted CD8 T cell neoepitope SIYRYYGL (designated as BP-SIY). This tumor model exhibits modest T cell infiltration and limited responses to ICB (Spranger et al., 2015), and expression of the SIY neoepitope does not alter tumor growth or the TME compared with parental *Braf*^*V600E*^/*Pten*^*fl/fl*^ tumors (Spranger et al., 2017). Twenty-five days post tamoxifen application, we initiated the 1X AIP +ICB therapy and found that the treatment significantly controlled tumor growth, while untreated lesions progressed to large tumor masses (Figure 1K). A limited exploration of alternate clinically relevant components in the priming regimen suggested that the anti-PD-1 in AIP can be replaced with anti-CTLA-4, whereas switching “A” or “I” with anti-CTLA-4 elicited decreased therapeutic efficacy depending on the tumor model (Figures S2C–S2F). Many combination immunotherapies suffer from unacceptable toxicities. However, 1X AIP treatment induced no significant changes in animal behavior, weight loss, or significant acute elevation of serum liver enzymes (Figures S2G and S2H). Altogether, 1X AIP appears to be an effective and safe combination treatment for enhancing responsiveness to ICB in multiple tumor models.

### Single-dose AIP therapy triggers rapid tumor antigen presentation

**Figure 2.**
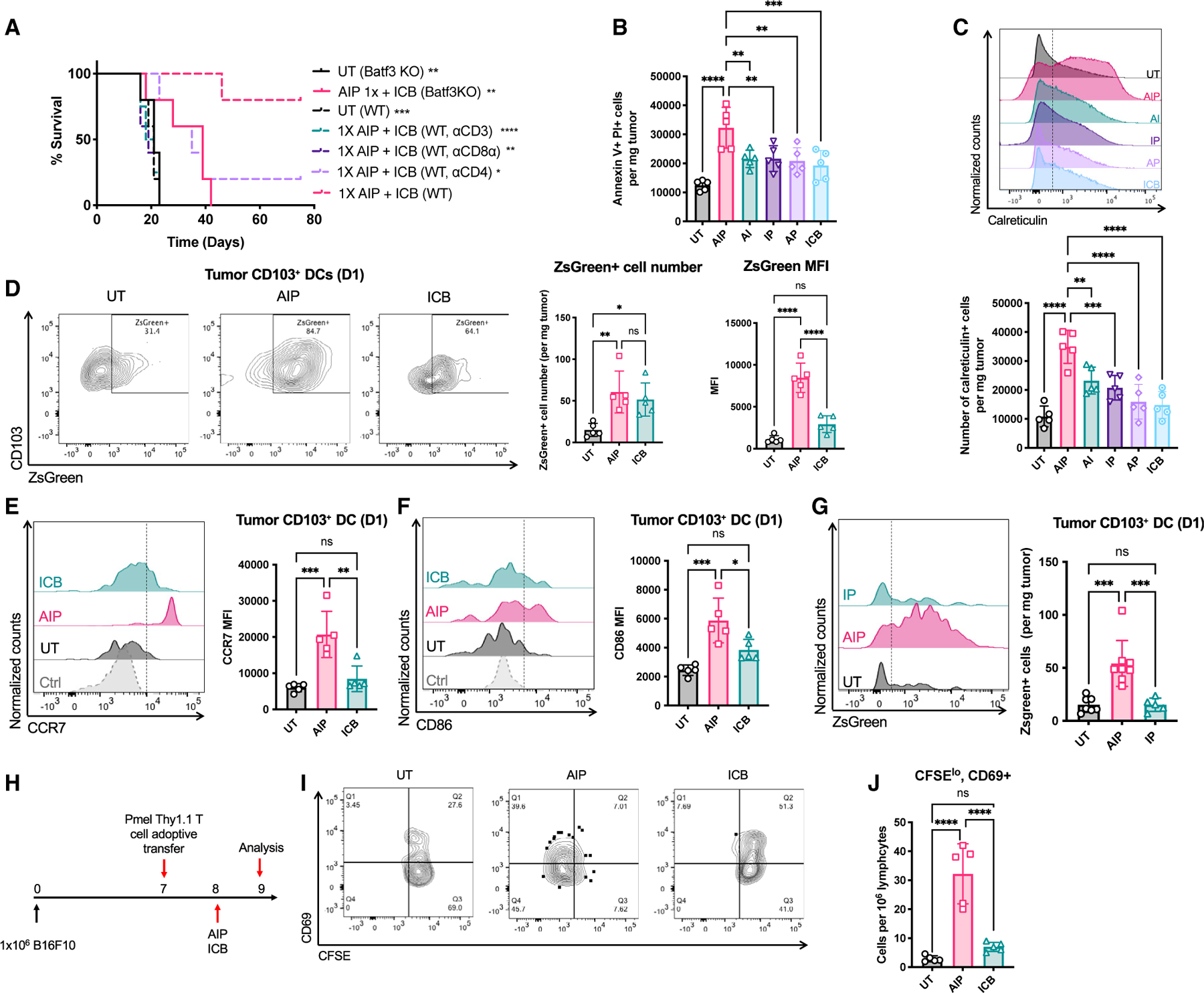
AIP immunotherapy activates antigen presentation in tumor-draining lymph nodes and promotes tumor-specific T cell activation (A) C57BL/6J mice bearing B16F10 tumors were left UT or treated with 1X AIP followed by ICB as in Figure 1G in the presence of depleting antibodies against CD3, CD8⍺, or CD4; shown is survival over time. Also shown is survival of tumor-bearing *Batf3*^−/−^ mice left UT or treated with 1X AIP + ICB. (B and C) C57BL/6J mice bearing B16F10 tumors were left UT or treated with 1X AIP followed by ICB as in Figure 1G. (B) Quantification of apoptotic tumor cells at 1 day post treatment. (C) Representative flow cytometry plots of cell surface calreticulin exposure on tumor cells 1 day post treatment. (D–G) C57BL/6J mice bearing ZsGreen-B16F10 tumors were left UT or treated with 1X AIP followed by ICB as in Figure 1G. (D) Quantification and representative flow cytometry plots of ZsGreen uptake by CD103^+^ DCs in tumors. (E and F) Quantification of ZsGreen^+^CD103^+^ DCs in the tumor and the expression of CD86 and CCR7 by those cells. (G) Quantification of ZsGreen^+^ CD103^+^ DCs in the tumor. Representative histogram plots of ZsGreen^+^ cDC1s in the tumor are also shown. (H–J) C57BL/6J mice bearing B16F10 tumors were injected with CFSE-labeled pmel T cells 1 day prior to 1X AIP treatment followed by flow cytometry analysis of TDLNs 1 day later. (H) Experiment timeline. (I and J) Representative flow cytometry plots (I) and quantification of activated proliferating pmel T cells in tumors (J). Throughout, n = 5 animals/group and shown are data from one representative of at least two independent experiments. *p < 0.05; **p < 0.01; ***p < 0.001; ****p <0.0001 versus AIP by log rank test in (A) and pairwise comparison by one-way ANOVA with the Tukey post test. All error bars are SD. See also Figure S3.

The development of anti-tumor T cell responses led us to assess the role of T cells and DCs in the response to 1X AIP therapy in the B16F10 model. Depletion of CD3^+^ T cells, CD4^+^ cells, or CD8^+^ cells all led to drastic reductions in therapeutic efficacy (Figure 2A; Figure S3A). Treatment of *Batf3*^−*/*−^ mice lacking cross-presenting DCs required for CD8^+^ T cell priming extended survival of tumor-bearing animals, but none rejected their tumors (Figure 2A).

We next sought to determine whether AIP treatment induces tumor antigen release and acquisition by DCs. One day post treatment with AI, IP, AP, or ICB, the number of apoptotic tumor cells was doubled compared with untreated tumors, but tumor cell death was increased 3-fold following treatment with AIP, and these cells exposed calreticulin that promotes phagocytosis (Chao et al., 2010) (Figures 2B and 2C). Using B16F10 cells expressing the stable fluorescent protein ZsGreen to enable tracking of antigen acquisition by DCs (Matz et al., 1999), we found a substantial increase in the level of tumor antigen uptake in intratumoral CD103^+^ DCs within 1 day of AIP treatment compared with untreated or ICB-treated tumors (Figure 2D). These cells also had higher expression of the costimulatory receptor CD86 and lymph node homing receptor CCR7 (Figures 2E and 2F). Comparing responses of ZsGreen-B16F10 tumors treated with a single dose of AIP versus IP, we found that the striking antigen uptake by cDC1s occurred only in the presence of the 2.5F-Fc antibody surrogate (Figure 2G). Adoptive transfer of pmel TCR-transgenic T cells that recognize the melanoma antigen gp100 into tumor-bearing mice just prior to therapy revealed that AIP induced antigen presentation and activation of tumor-specific T cells in TDLNs by 1 day following treatment (Figures 2H–2J). These data indicate that a single dose of AIP is able to rapidly induce tumor antigen release, which is captured by activated DCs to initiate T cell priming in TDLNs.

### NK cells and macrophages play a critical role in tumor rejection following 1X AIP therapy

We hypothesized that rapid tumor cell death induced following AIP administration was mediated at least in part by FcR-expressing macrophages and NK cells responding to the “A” component.

**Figure 3.**
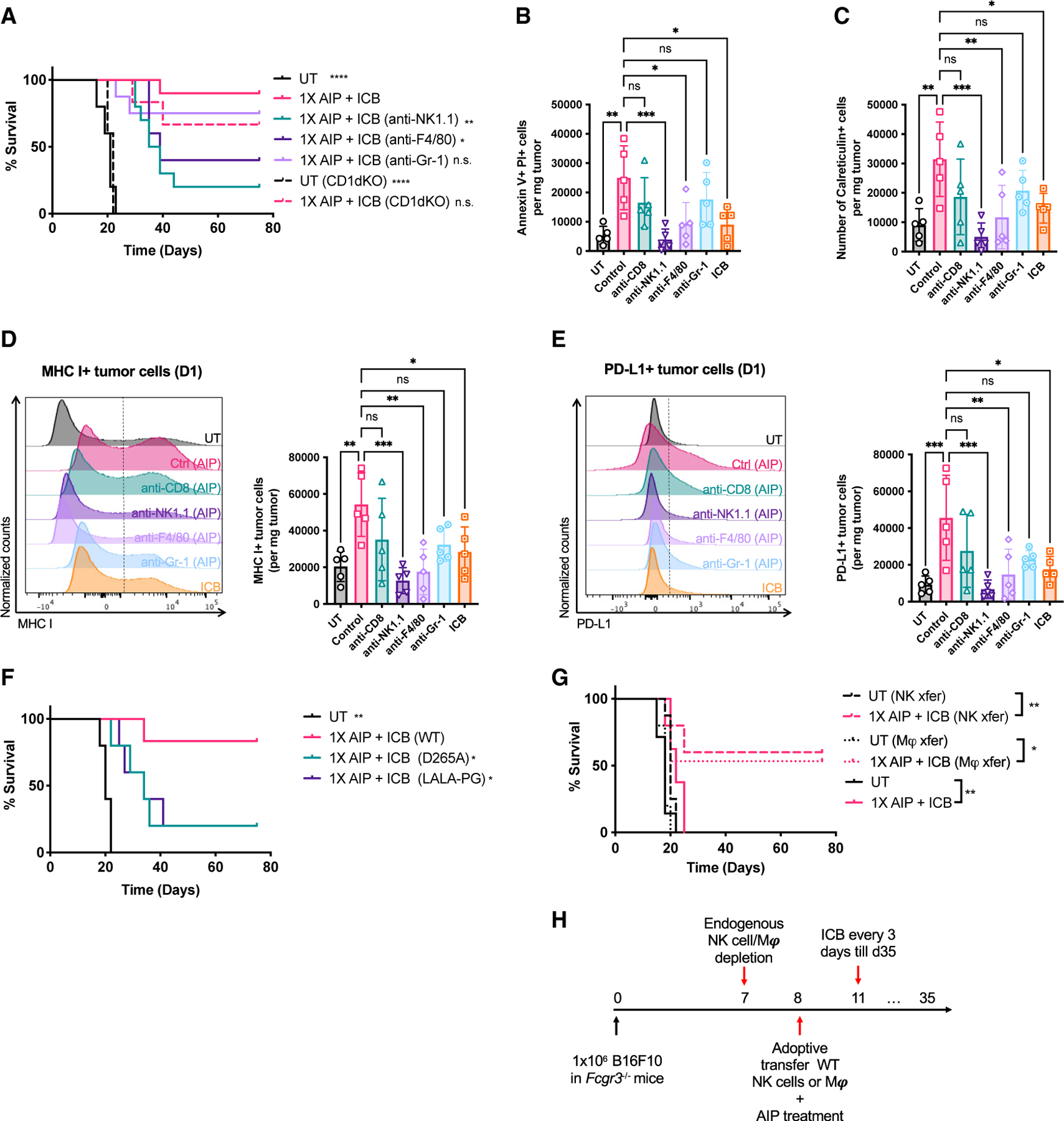
NK cells and macrophages play an important role in initial killing of tumor cells and tumor antigen delivery to DCs (A) Survival of B16F10 tumor-bearing mice over time with depleting antibodies for NK cells and macrophages injected 1 day before 1X AIP treatment (n = 5 mice/group, from one representative of two independent experiments). (B and C) Quantification of apoptotic (B; Annexin V^+^, PI^+^) and surface calreticulin^+^ (C) tumor cells in UT, AIP-treated tumors with depleting antibodies for CD8^+^ T cells, NK cells, and macrophages 1 day before treatment. (D and E) Representative flow cytometry histograms and quantification of MHC class I (D) and PD-L1 (E) expression on tumor cells (defined as live, CD45^−^ and TA99^+^ cells) from mice 1 day post treatment as in (B) and (C). (F) Survival over time following 1X AIP + ICB treatment in mice bearing B16F10 tumors (n = 5 mice/group), where D265A or LALA-PG mutations were introduced to the Fc portion of 2.5F-Fc. (G and H) FcγRIII-deficient mice (n = 8–10 animals/group, pooled from two independent experiments) bearing B16F10 tumors were depleted of endogenous NK cells or macrophages 1 day prior to treatment with 1X AIP + ICB. One group of mice received adoptive transfer of 1 × 10^6^ WT NK cells (dashed lines), and another group received 3–4 × 10^6^ WT macrophages (dotted lines) injected on day 0. Tumor-bearing FcγRIII-deficient mice treated with or without 1X AIP + ICB (with no depletion or adoptive transfer) were used as a control (solid lines) Shown is survival of these mice over time (G) and the experiment timeline (H). *p < 0.05; **p < 0.01; ***p < 0.001; ****p < 0.0001 versus 1X AIP + ICB by log rank test in (A), (F), and (G) and by one-way ANOVA followed by Dunnett post test in (B) and (C) and (D) and (E). All error bars are SD. See also Figure S3.

Notably, the therapeutic efficacy of 1X AIP was greatly reduced when either NK cells or macrophages, but not neutrophils, were depleted prior to treatment (Figure 3A; Figure S3B). The lack of contribution from neutrophils is consistent with their limited infiltration into the tumors (Figure S3C), in contrast with what we observed with full AIPV treatment (Moynihan et al., 2016). As NK1.1 upregulation by macrophages has been reported (Steiger et al., 2015), we evaluated the expression of NK1.1 and F4/80 in tumor-infiltrating macrophages and NK cells, respectively, to confirm that the depleting antibodies used here were not potentially removing unintended cellular targets. We found minimal expression of NK1.1 on macrophages or F4/80 on NK cells and no significant difference between treated and untreated conditions (Figure S3D). Similarly, F4/80 has been reported to be expressed on activated CD8^+^ T cells in some conditions (Lin et al., 2003), but we observed no expression of F4/80 on tumor-infiltrating CD8^+^ T cells in treated or untreated tumors (Figure S3D) (Lin et al., 2003). Furthermore, neither CD1d-restricted NK T (NKT) cells nor “late” NK cell depletion (3 days post AIP treatment) had a significant impact on the efficacy of 1X AIP therapy (Figure 3A; Figure S3E). Depletion of NK cells or macrophages, but not T cells or neutrophils, also led to a statistically significant reduction in immediate tumor cell death following AIP (Figures 3B and 3C). 1X AIP induced major histocompatibility complex class I (MHC class I) and PD-L1 upregulation on cancer cells within 1 day, a response that was substantially reduced if NK cells or macrophages were depleted (Figures 3D and 3E). We next assessed whether rapid NK cell- and macrophage-dependent tumor-cell killing was mediated by the antibody surrogate. Introduction of a D265A mutation in the Fc domain of 2.5F-Fc to ablate binding of the molecule with FcγRs (Baudino et al., 2008) led to a loss of rapid tumor cell death following AIP and substantial reductions in treatment efficacy (Figure 3F; Figure S3F). The upregulation of MHC class I and PD-L1 on cancer cells was also lost when D265A or LALA-PG mutations were introduced in the 2.5F-Fc antibody surrogate (Figures S3G and S3H).Treatment with 2.5F-Fc bearing LALA-PG mutations in the Fc domain to block both FcγR binding and complement activation (Lo et al., 2017) did not further blunt efficacy, suggesting complement does not play a major role. FcγRIII is a key activating FcγR that mediates antibody-dependent cellular cytotoxicity (ADCC) by NK cells (Mandelboim et al., 1999) and is also expressed on macrophages (Kinder et al., 2015). FcγIIIR deficiency completely abolished the therapeutic efficacy of 1X AIP treatment, further supporting a requirement for the Fc domain of 2.F-Fc and ADCC for the response to treatment (Figure 3G). To test the importance of FcRs on macrophages and NK cells specifically, we used an adoptive transfer approach, with antibody-mediated depletion of endogenous NK cells/macrophages from tumor-bearing *Fcgr3*^−*/*−^ mice followed by intravenous (i.v.) transfer of wild-type (WT) NK cells or macrophages just before 1X AIP treatment (Hagemann et al., 2008; Tai et al., 2013) (Figure 3H). Adoptive transfer of either WT NK cells or macrophages into *Fcgr3*^−/−^ mice restored the response rate to 1X AIP treatment to 50%–60%, indicating an essential role for functional FcγRs on NK cells and macrophages in the first few days of the treatment (Figure 3G).

CD8α antibody-mediated depletion showed a trend toward some impact on tumor antigen uptake by CD103^+^ DCs, which could be an indirect effect of removing CD8α^+^ DCs by the antibody, but transient intratumoral depletion of T cells (Besançon et al., 2017) in the first few days of treatment did not alter long-term survival in response to 1X AIP therapy (Figure S3I). Thus, while T cells are critical for ultimate tumor rejection, they are not important for early events following 1X AIP priming. Rather, NK cells and macrophages played a dominant role in the early events leading to the efficacy of AIP priming followed by checkpoint blockade.

### AIP induces major transcriptional changes in tumor-infiltrating NK cells and macrophages

**Figure 4.**
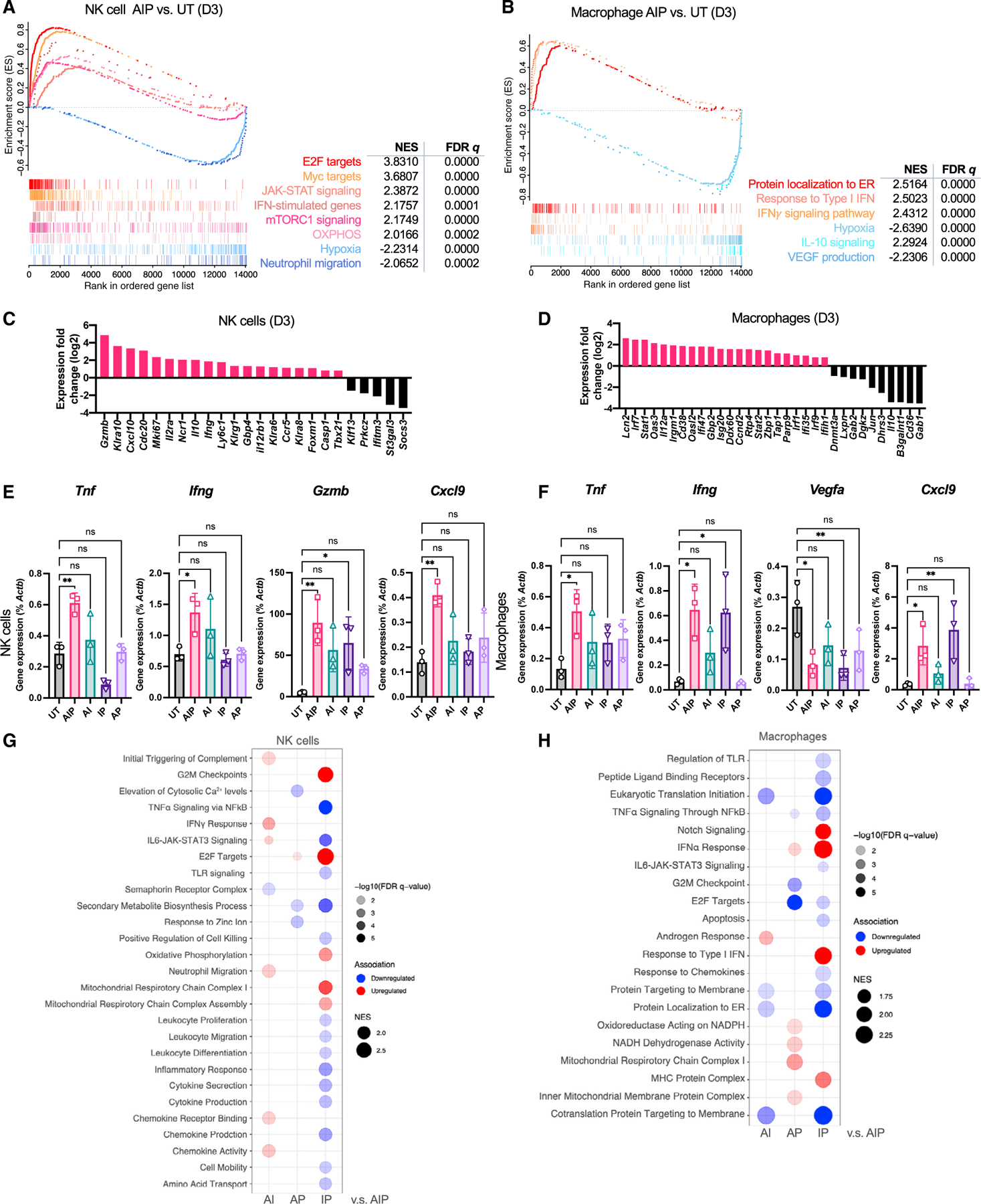
Intratumoral NK cells and macrophages are rapidly reprogrammed by AIP treatment Mice bearing B16F10 tumors (n = 3–5 animals/group) were left UT or treated with 1X AIP + ICB as in Figure 1G, followed by sorting of macrophages or NK cells and bulk RNA sequencing 3 days post treatment. (A) Gene set enrichment analysis (GSEA) for NK cells in AIP versus UT tumors. (B) GSEA for macrophages in AIP versus UT tumors. (C and D) Expression of NK cell (C)-specific and macrophage (D)-specific genes related to their activation status and subtypes in tumor-infiltrating NK cells and macrophages isolated from AIP-treated or UT tumors 3 days post treatment. (E and F) Expression of example genes as percentage of *Actb* from NK cell (E) and macrophage (F) samples determined by qPCR. *p < 0.05; **p < 0.01; ****p < 0.0001 versus UT by one-way ANOVA with Dunnett post test. (G and H) GSEA of NK cells (G) and macrophages (H) across dual-combination treatment groups compared with those from AIP-treated mice. Red indicates upregulation and blue indicates downregulation. Circle size is proportional to NES, and color shade is proportional to false discovery rate *q* value as indicated (NES > 1.5 and *q* value < 0.01). All error bars are SD. See also Figure S4.

Given the important role played by NK cells and macrophages in the response to AIP therapy, we next carried out RNA sequencing (RNA-seq) to determine how stimulation by AIP, AI, IP, or AP combinations altered transcriptional programs in these cells. Mice bearing B16F10 tumors were treated with AIP or the two-component subcombinations, and 3 days later NK cells or macrophages were sorted for bulk RNA-seq. Gene signature enrichment analysis revealed that AIP-treated NK cells and macrophages were strongly activated, as indicated by the positive engagement of IFN-γ and type I IFN signaling (Figures 4A and 4B). Compared with NK cells from untreated tumors, AIP-treated cells showed upregulation of effector function genes (e.g., *Gzmb* and *Ifng*) and downregulation of the cytokine signaling suppressor *Socs3* (Palmer and Restifo, 2009) (Figure 4C). Genes that are known to be upregulated early after infections, such as *Ncr1*, *Klra8*, and *Il12rb1*, were also increased in AIP-treated NK cells (Bezman et al., 2012). Interestingly, genes that are usually upregulated in late/sustained effector NK cells, such as *Mki67* and *Foxm1*, and genes related to long-lived memory NK cells, such as *Casp1*, *Klra6*, *Klra10*, *Ly6c1*, and *Klrg1*, were also higher in AIP-treated NK cells (Gautier et al., 2012; Sun et al., 2009) (Figure 4C). An increased presence of memory NK cells in the TME in AIP-treated tumors was validated through flow cytometry (Figures S4A and S4B). Compared with the untreated condition, AIP-treated NK cells were also more metabolically active through increases in mTORC1 and MYC signaling and oxidative phosphorylation (Figure 4A), which are known to be associated with elevated NK cell proliferation, IFN-γ production, and cytotoxicity (Donnelly et al., 2014; Gotthardt et al., 2016). In macrophages, AIP induced upregulation of many IFN-regulated genes, such as *Ifi35*, *Ifi47*, *Ifih1*, *Isg20*, *Irgm1*, *Irf1*, *Irf9*, *Oas3*, *Oasl2*, *Rtp4*, *Tap1*, and *Zbp1*, together with pro-M1 factors such as *Il12a*, coincident with higher expression of signal transducer and activator of transcription 1 and 2 (*Stat1* and *Stat2*) (Figure 4D). Increased levels of *Cd38* and *Parp9* (Iwata et al., 2016), which have been shown to mark activated M1-like macrophages (Amici et al., 2018; Jablonski et al., 2015), were also observed (Figure 4D). On the other hand, gene signatures that have been shown to be associated with M2-like macrophages (Orecchioni et al., 2019), such as *Il10*, *Cd36* (Vats et al., 2006), *B4galnt1*, *Dgkz*, *Gab1* (Guo et al., 2017), and *Jun*, were downregulated in the AIP treatment group, indicating a pro-inflammatory signature in these cells. We confirmed upregulation of a subset of effector genes in these cells (e.g., *Tnf*, *Ifng*, *Gzmb*, and *Nos2)* by qPCR (Figures 4E and 4F; Figures S4C and S4D). Cross-comparison of genes and pathways upregulated in NK cells and macrophages from mice treated with AI, AP, or IP versus AIP revealed the largest differential between AIP and IP, suggesting that the antibody surrogate exerts the strongest effect on both of these innate cell populations (Figures 4G and 4H). Interestingly, little overlap was observed in pathways regulated by “A,” “I,” or “P,” suggesting distinct roles for each component in the treatment regimen.

Intriguingly, both NK cells and macrophages showed shifts in gene expression following AIP treatment related to the tumor vasculature and cell recruitment: Both innate populations downregulated genes associated with hypoxia, and macrophages downregulated VEGF production associated with neo-angiogenesis (Figures 4A, 4B, and 4F). In parallel, expression of CXCL9 was upregulated in both cell types (Figures 4E and 4F). Thus, AIP triggered a rapid reprogramming of intratumoral NK and macrophage states.

### NK cell- and macrophage-dependent recruitment of lymphocytes to AIP-treated tumors

**Figure 5.**
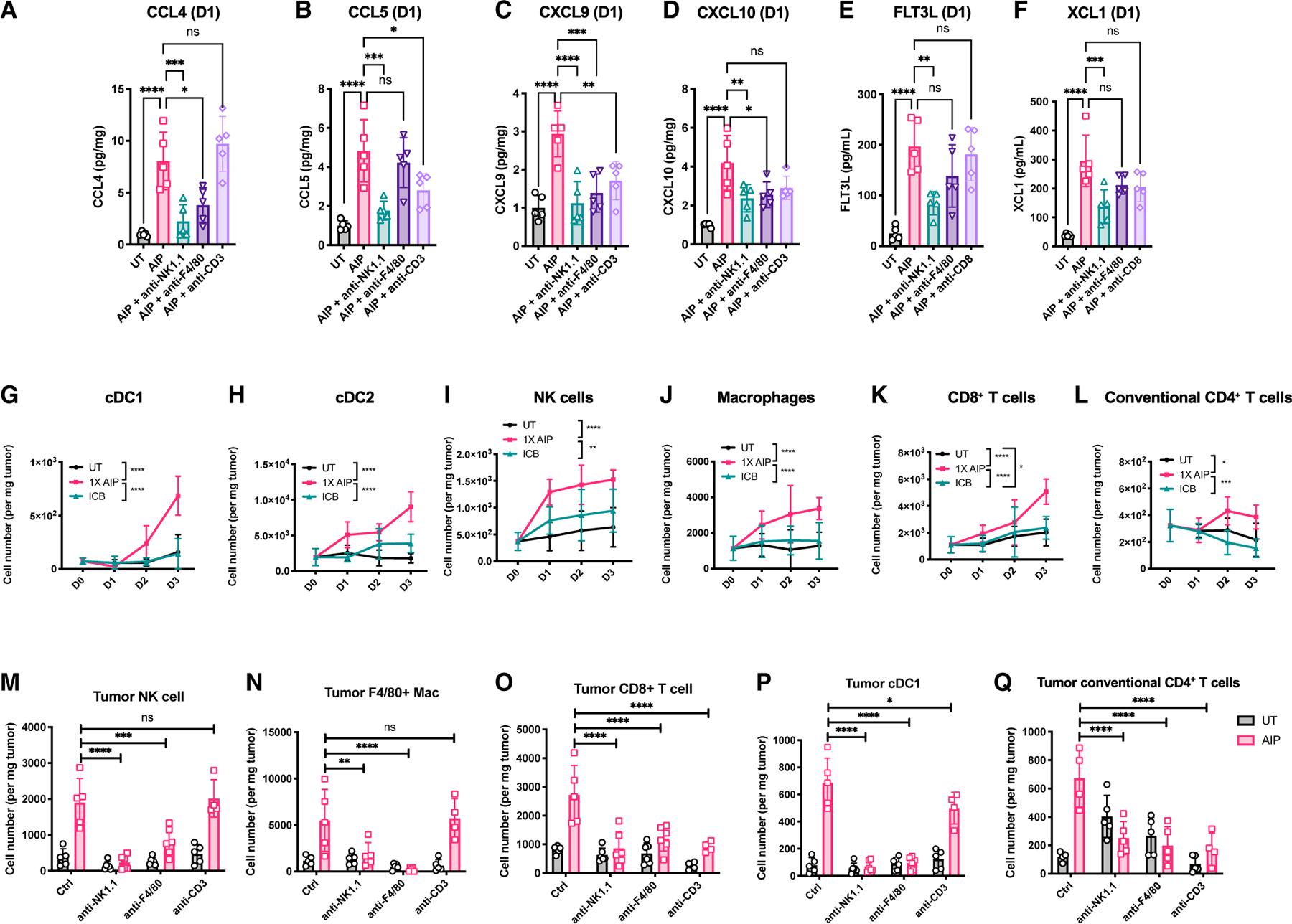
NK cells and macrophages govern immune infiltration of tumors following 1X AIP therapy Mice bearing B16F10 tumors (n = 5 animals/group) were left UT, treated with ICB only, or treated with 1X AIP + ICB as in Figure 1G, in the presence or absence of depleting antibodies against NK1.1, F4/80, or CD3. (A–F) Levels of the indicated chemokines and cytokines in tumor lysates were measured by Luminex (A–D) or standard ELISA (E and F) 1 day post the first dose of AIP treatment. (G–L) Tumors were isolated 1, 2, or 3 days post treatment and were analyzed by flow cytometry. Shown are quantifications of the total number of tumor-infiltrating CD103^+^ DCs (G), CD11b^+^ DCs (H), NK cells (I), macrophages (J), CD8^+^ T cells (K), and conventional CD4^+^ T cells (L). (M–Q) Quantification of tumor-infiltrating NK cells (M), macrophages (N), CD8^+^ T cells (O), CD103^+^ DCs (P), and conventional CD4^+^ T cells (Q) in mice with indicated treatments (n = 4–5 mice per group from 2 independent experiments). *p < 0.05; **p < 0.01; ***p < 0.001; ****p < 0.0001 versus 1X AIP + ICB by two-way ANOVA with the Holm-Šídák test in (G) to (Q) and one-way ANOVA with Dunnett post test in (A) to (F). All error bars are SD. See also Figure S4.

Similar to what we observed following AIPV treatment, AIP administration elicited rapid upregulation of a broad range of chemokines in tumors as well as a dramatic upregulation of the DC-expanding cytokine FLT3L (Guermonprez et al., 2013) (Figures 5A–5F; Figures S4E–S4G). FLT3L levels also increased in the serum of AIP-treated mice, consistent with an expansion of CD11c^+^ DCs even in non-TDLNs (Figures S4H and S4I). Depletion of NK cells, macrophages, or T cells revealed that NK cells played the biggest role in driving production of these factors, although all 3 cell types contributed (Figures 5A–5F; Figures S4E–S4G).

Consistent with the rapid upregulation of chemokines in treated tumors, 1X AIP induced the recruitment of DCs, NK cells, macrophages, and T cells over the first 3 days post injection (Figures 5G–5L). This early accumulation of lymphocytes was predominantly new recruitment of cells to the tumor and not local proliferation, as blocking lymphocyte egress from lymphoid organs with FTY720 substantially lowered the infiltration of all of these cell types except macrophages (Figures S5A–S5F). To directly assess the role of NK cells, macrophages, and T cells in immune cell recruitment to AIP-treated tumors, we depleted each cell type starting 1 day prior to therapy and analyzed TILs 1 or 3 days after AIP dosing. Interestingly, we saw that depletion of NK cells led to a decrease in the number of intratumoral macrophages, and vice versa. At the same time, a significant reduction in the cellularity of intratumoral cDC1, CD8^+^, and conventional CD4^+^ T cells was observed upon depletion of either NK cells or macrophages (Figures 5M–5Q). By contrast, T cell depletion showed limited impact on the number of intratumoral NK cells, macrophages, or cDC1s (Figures 5M–5Q). Thus, NK cells and macrophages govern the infiltration of multiple immune cell line-ages following 1X AIP treatment.

### AIP treatment induces rapid tumor vasculature remodeling

**Figure 6.**
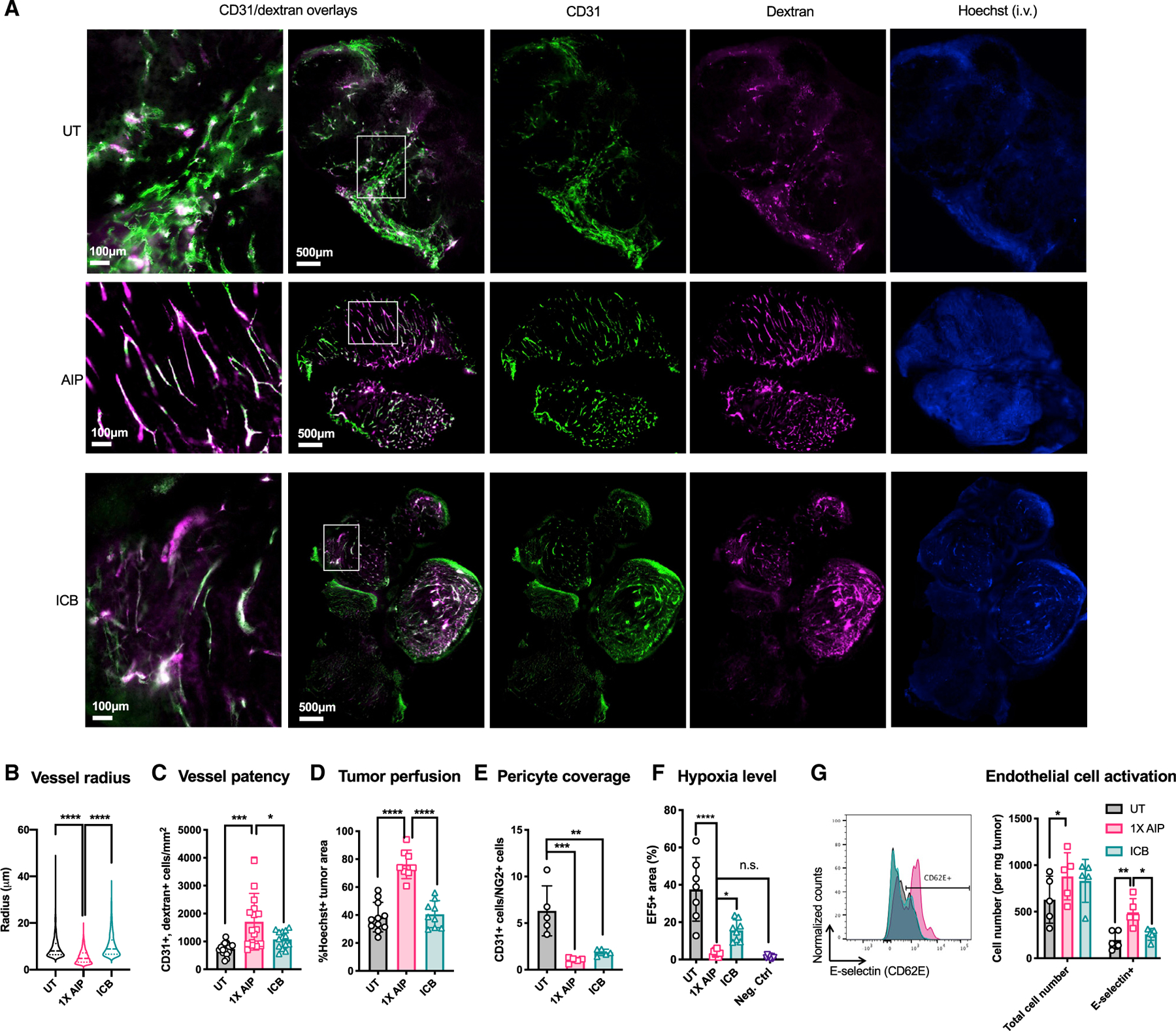
1X AIP treatment induces tumor blood vessel normalization Mice bearing B16F10 tumors (n = 5–6 animals/group, pooled from two independent experiments) were left UT, treated with ICB alone, or treated with 1X AIP + ICB as in Figure 1G. Three days later, 70 kDa fluorescent dextran and Hoechst dye were injected i.v., and animals were sacrificed 10 min later for tumor harvest and immunohistochemistry. (A) Representative histological sections of UT, AIP-treated, and ICB-treated tumors. Green, CD31 staining; pink, dextran; blue, Hoechst staining. Scale bars are 100 μm and 500 μm for the first column of and the rest of the images, respectively. (B) Quantification of the radius of CD31^+^ blood vessels from CUBIC-R-cleared B16F10 tumors 3 days post AIP and ICB treatment or left UT. (C and D) Quantifications of CD31^+^dextran^+^ cells (C) and the fraction of Hoechst^+^ areas (D) shown in (A). (E) Quantification of the ratio of endothelial cells to pericytes by flow cytometry from UT, AIP-treated, or ICB-treated B16F10-bearing mice. (F) Quantification of the hypoxia levels in tumors marked using the hypoxia-detecting molecule EF5 in mice treated as described in (D). (G) Quantification of total number and activated intratumoral endothelial cells in mice treated as described in (D). *p < 0.05; **p < 0.01; ***p < 0.001; ****p < 0.0001 versus UT or 1X AIP + ICB by one-way ANOVA with Dunnett post test (B–F) and two-way ANOVA with Tukey post test (G). All error bars are SD. See also Figures S5 and S6.

Given the dramatic enhancement in immune cell recruitment induced by AIP priming and changes in hypoxia gene signatures in innate cells following treatment, we hypothesized AIP therapy might directly alter the tumor vasculature in ways promoting anti-tumor immunity. Staining of the endothelial cell marker CD31 three days post AIP treatment revealed a significant reduction in the radius and size variation of vessels in AIP-treated tumors compared with untreated or ICB-treated tumors (Figures 6A and 6B). Colocalization of CD31 staining with fluorescent dextran injected just prior to sacrifice suggested an increase in the proportion of patent vessels compared with untreated tumors or tumors receiving checkpoint blockade alone (Figure 6A, colocalization appears white; Figure 6C). Furthermore, Hoechst dye injected i.v. into AIP-treated mice 10–15 min prior to sacrifice was found distributed throughout the entire tumor cross section, in contrast with patchy intensity observed in histological sections collected from untreated tumors or tumors treated with ICB alone, suggesting enhanced perfusion of AIP-treated tumors (Figures 6A and 6D). Pericytes, which support the stability and function of vascular endothelial cells (ECs), are reduced in number and become malfunctional in tumors (Chao et al., 2010). Using Neural/glial antigen 2 (NG2) to identify pericytes, we found that compared with untreated controls, AIP-treated tumors showed a substantially higher percentage of pericytes covering ECs and a lower ratio of CD31^+^ cells/NG2^+^ cells as determined by immunofluorescent staining and flow cytometry, suggesting a more mature vascular network stabilized by pericytes in these tumors (Figure 6E; Figures S6A and S6B). Furthermore, intratumoral hypoxia, determined by staining tumors with the hypoxia-sensitive molecule EF5, was 11-fold lower in AIP-treated tumors than untreated tumors and 4.5-fold lower than in tumors treated with ICB alone (Figure 6F; Figure S6C). Finally, flow cytometry analysis revealed that both AIP and ICB induced a modest increase in total number of vascular ECs, but that ECs in the 1X AIP treatment group also upregulated expression of key adhesion molecules promoting immune infiltration, such as E-selectin (CD62E) (Figure 6G). These changes in the TME do not appear to relate to direct effects of the 2.5F-Fc molecule on tumor ECs, as AIP therapy using the TYRP-1-specific TA99 antibody instead of 2.5F-Fc elicited similar changes in B16F10 tumors (Figure S7A).

**Figure 7.**
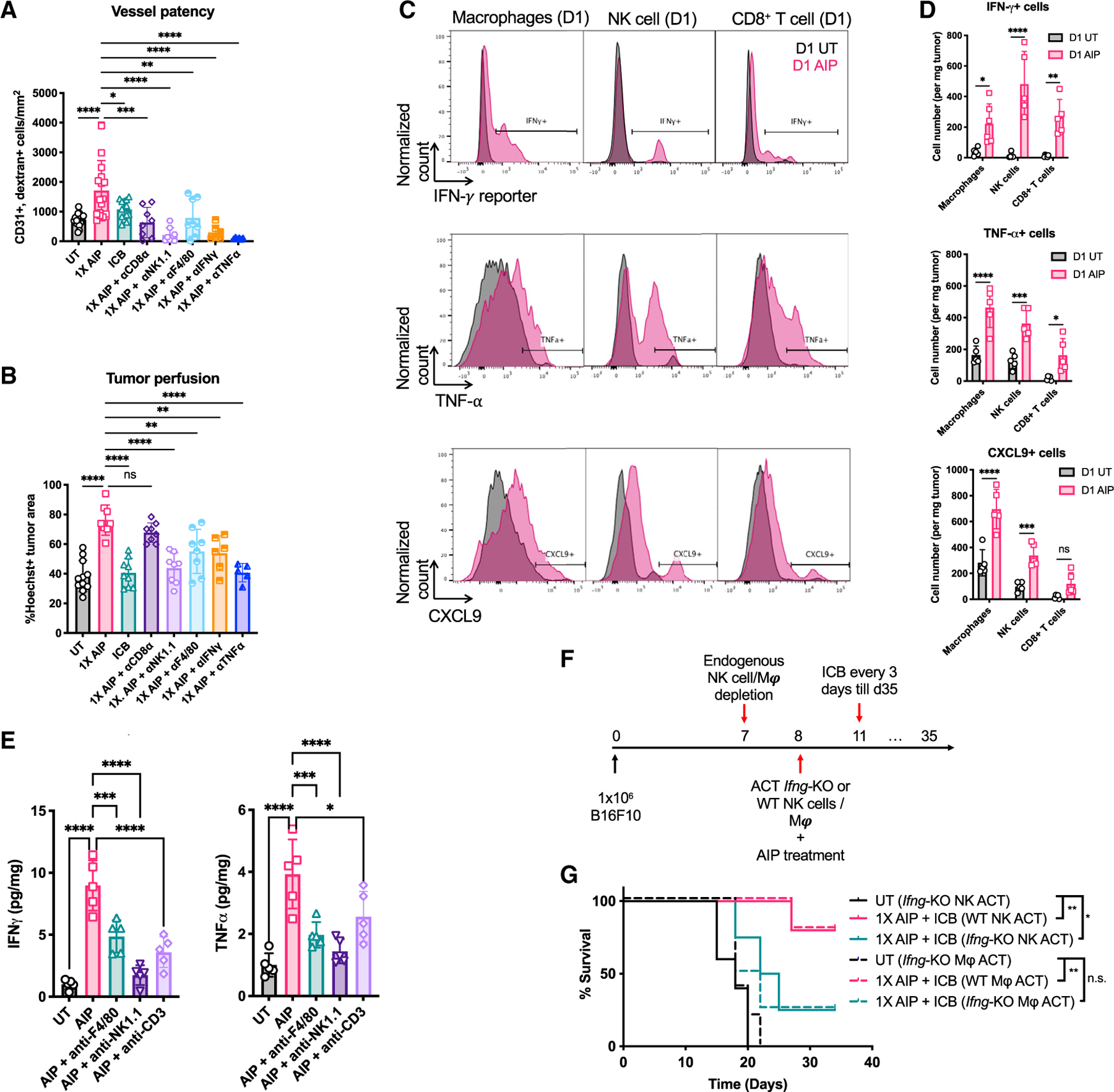
IFN-γ produced by macrophages and NK cells is required for the therapeutic efficacy of 1X AIP (A–E) Mice bearing B16F10 tumors (n = 5–6 animals/group, pooled from at least two independent experiments) were left UT, treated with ICB alone, or treated with 1X AIP + ICB as in Figure 1G. Some groups received depleting antibodies against NK cells, macrophages, or IFN-γ, TNF-⍺ beginning 1 day prior to treatment as indicated. (A and B) Shown are quantifications of vessel patency (CD31^+^dextran^+^ cells/mm^2^; A) and tumor perfusion (Hoechst^+^ % area; B) levels in tumor sections. (C and D) B16F10-bearing IFN-γ reporter mice were UT or treated with 1X AIP as in Figure 1G. Tumors were collected 1 day post treatment. The numbers of IFN-γ-, TNF-⍺- and CXCL9-secreting macrophages, NK cells, and CD8^+^ T cells were determined by flow cytometry. (E) Quantification of IFN-γ and TNF-⍺ levels in B16F10-bearing mice 1-day post AIP treatment in the presence of indicated depleting antibodies. (F and G) Mice bearing B16F10 tumors (n = 5 animals/group) were depleted of endogenous NK cells or macrophages one day prior to treatment with 1X AIP + ICB. On the day of treatment, animals received adoptive transfer of WT or IFN-γ-deficient macrophages (3–4 × 10^6^) or NK cells (1 × 10^6^). Shown is the experiment timeline (F) and survival of these mice over time (G). Throughout, *p < 0.05; **p < 0.01; **p < 0.001; ****p < 0.0001 determined by one-way ANOVA with Dunnett post test (A, B, and E), two-way ANOVA followed by Holm-Šídák test (D), and log rank test (G). All error bars are SD. See also Figure S7.

We next sought to determine which immune cells were critical for the vasculature changes induced by AIP treatment. Depletion of CD8^+^ T cells, macrophages, or NK cells reduced the density of patent vessels (Figure 7A; Figure S7B). By contrast, increased perfusion of tumors as revealed by Hoechst staining and increased pericyte coverage of vessels following treatment was dependent on macrophages and NK cells, but not T cells (Figure 7B; Figures S6A and S6B). Hence, AIP priming promotes a more normalized tumor vascular network through the combined activity of innate and adaptive immune cells.

### Cytokine production by NK cells and macrophages facilitates vascular normalization and is critical for the therapeutic efficacy of AIP treatment

Both IFN-γ and TNF-α have been shown to exert effects on not only hematopoietic cells but also tumor vascular ECs and/or stromal fibroblasts, altering tumor vasculature to promote anti-tumor immunity (Johansson et al., 2012; Lu et al., 2009; Tian et al., 2017). Given the immediate upregulation of IFN-γ and TNF-α following 1X AIP (Figures S2A and S4C), we next assessed whether these cytokines might be important drivers of the therapeutic effects of AIP priming. Neutralization of IFN-γ or TNF-α largely diminished vessel patency and tumor perfusion in the AIP treatment group (Figures 7A and 7B). Using an IFN-γ reporter mouse (Reinhardt et al., 2015) and intracellular staining as complementary methods, we found that AIP treatment elicited a substantial increase in the number of IFN-γ^+^ NK cells, macrophages, and T cells in tumors within 1 day, and induced CXCL9 and TNF-α expression in all three populations of effector cells (Figures 7C and 7D; Figures S7C and S7D). Consistent with these findings, depletion of any of these cell types lowered the overall levels of TNF-α and IFN-γ protein present in treated tumors (Figure 7E). Thus, both T cells and innate cells contribute key cytokines promoting the vascular normalization phenotype observed in AIP-treated tumors. However, IFN-γ production by macrophages and NK cells was essential to the success of the treatment, as depletion of endogenous NK cells or macrophages followed by adoptive transfer of IFN-γ-deficient innate cells into WT B16F10 tumor-bearing mice resulted in compromised therapeutic efficacy (Figures 7F and 7G). Thus, cytokine production by NK cells and macrophages plays a key role in chemokine and vascular changes promoting immune infiltration into initially cold melanoma tumors, and IFN-γ produced by these cells is critical for the striking therapeutic efficacy of AIP priming.

## DISCUSSION

ICB therapy removes inhibitory signaling in tumor-infiltrating T cells to achieve clinical benefit, but “cold” tumors lacking pre-existing infiltration are often unresponsive to ICB (Colli et al., 2016; Snyder et al., 2014; Topalian et al., 2012; Tumeh et al., 2014). Despite vigorous exploration of therapeutic approaches that could transform “cold” tumors into the “hot” lymphocyte-infiltrated state, this remains an important immunotherapeutic hurdle believed to be limiting responses observed with checkpoint blockade (Gajewski, 2015). There is mounting evidence suggesting treatments that recruit innate immune effector mechanisms can rapidly alter the TME via induction of tumor cell death and inflammation of the microenvironment, thus forming a feed-forward loop to boost T cell recruitment, function, and proliferation (Arnould et al., 2006; Corrales et al., 2015; Lee et al., 2009; Moynihan and Irvine, 2017; Park et al., 2010). Building on earlier work where we discovered a potent combination treatment that activated both innate and T cell responses against tumors (Moynihan et al., 2016), here we show that a variety of tumor models exhibiting weak responses to ICB can be cured by it following a “priming” dose of AIP therapy comprised of an anti-tumor antibody surrogate, long half-life IL-2, and anti-PD-1. All three components in AIP were required to achieve the maximum therapeutic efficacy in recalcitrant tumor models.

Therapeutic strategies aiming to induce tumor antigen release, stimulate APCs and intratumoral inflammation, or promote vascular normalization have been reported to exhibit synergy with checkpoint blockade (Mullins et al., 2019; Wang-Bishop et al., 2020; Wu et al., 2017). Notably, we find that AIP treatment activates NK cells and macrophages to promote all of these effects in tandem. Activated NK cells and macrophages mediated rapid tumor cell killing in the presence of the antibody surrogate through ADCC; the resulting tumor debris in immune complexes is known to effectively promote antigen uptake and activation of DCs (DiLillo and Ravetch, 2015). In tandem, AIP elicited major transcriptional changes in the effector functions of NK cells and pro-inflammatory “M1” macrophages, including increased IFN-γ and type I IFN signaling (Figures 4A–4D). Metabolic reprogramming of NK cells is reflected by the engagement of mTORC1 and OXPHOS pathways (Figure 4A), which has been shown to correlate with IFN-γ production and cytolytic activity in NK cells (Donnelly et al., 2014). Interestingly, AI seemed to activate stronger IFN-γ expression in NK cells than AIP (Figure 4G), which might result from Treg activation promoted by PD-1 blockade (Kamada et al., 2019; Tan et al., 2021). However, AIP therapy was ultimately much more effective for tumor rejection following subsequent ICB administration.

Recent studies have pointed to an important role for NK cells in mediating DC recruitment to tumors in the steady state through the production of chemokines (such as CCL4/5 and XCL1) and FLT3L to expand DC precursors (Barry et al., 2018; Böttcher et al., 2018). Here, we find that engaging NK cells and macrophages through an anti-tumor antibody and IL-2 (for NK cells) can therapeutically drive such chemokine and FLT3L production in the TME (Figures 5A, B, E, and F). Moreover, AIP-stimulated NK cells and macrophages not only acted as gatekeepers for DC infiltration but also mediated the recruitment of CD8^+^ T cells, CD4^+^ T cells, and additional NK cells and macrophages. This appeared to be a truly cooperative process as depletion of NK cells or macrophages individually led to a collapse of immune infiltration to near the baseline of untreated tumors. The loss of either NK cells or macrophages also resulted in a major change in the cytokine and chemokine profile of the TME, and eventually most animals succumbed to tumor outgrowth (Figure 3A; Figure S4C). These findings are consistent with studies showing that upon activation, NK cells produce macrophage-activating and -attracting factors, including IFN-γ (Duluc et al., 2009), CCL3 (Allen et al., 2017), GM-CSF (Louis et al., 2020), etc., while activated macrophages secrete cytokines such as IFN-γ (Pak-Wittel et al., 2013) and IL-12 and IL-15 (Lapaque et al., 2009) to attract and stimulate NK cells, forming a positive feedback loop. Consistently, we found that IFN-stimulated and proliferation-related genes and pathways were among the top enriched gene sets in NK cells and macrophages isolated from AIP-treated tumors.

Neutrophils are deemed as first-line responders to inflammation induced by pathogens or immunotherapies. Notably, neutrophils were found to be critical for therapeutic responses to the original full AIPV regimen (Moynihan et al., 2016), but were not as important in 1X AIP treatment (Figure 3A; Figure S3B). This difference may reflect a continuously increasing number of total and activated neutrophils in tumors evoked during AIPV treatment due to repeated dosing of IL-2, which has been shown to stimulate both human and mouse neutrophils (Assier et al., 2004; Donskov et al., 2006). However, in 1X AIP-treated tumors, the number of neutrophils increased 1 day after the AIP treatment, but subsequently reverted to baseline levels within 3 days (Figure S3C).

Perhaps most striking was the finding that AIP treatment induced substantial, rapid changes in the tumor vasculature by NK cells and macrophages, and to a lesser extent, T cells. Tumor vasculature is known to be defective in supporting immune infiltration through downregulation of adhesion molecules, reduction of oxygen availability and many other mechanisms (Salazar and Zabel, 2019). AIP induced significant increases in multiple measures of tumor vasculature normalization within 3 days of dosing, including decreased vessel radius, increased vessel patency and perfusion of the tumor, ameliorated hypoxia, and increased expression of adhesion receptors required for lymphocyte transmigration. These changes in the tumor were lost when IFN-γ or TNF-α were neutralized. Sustained production of IFN-γ has been reported to induce blood vessel destruction and necrosis that leads to tumor rejection (Briesemeister et al., 2011; Kammertoens et al., 2017), but we observed a slightly higher number of live ECs in AIP-treated mice (Figure 6G). However, in these animals, we did observe decreased *Vegfa* in macrophages (Figure 4F) and increased angiostatic factors *Cxcl9* and *Cxcl10* in both NK cells and macrophages (Figures 4E, 4F, 5C, and 5D), which are known to be regulated by IFN-γ. It has been shown that GTPase guanylate-binding protein 1 (GBP-1) in endothelial cells also mediates the angiostatic activities of IFN-γ (Guenzi et al., 2001; Lubeseder-Martellato et al., 2002). Hence, we expect IFN-γ may act both directly and indirectly toward vessel normalization following 1X AIP treatment. On the other hand, vasculature-targeted low-dose TNF-α has also been shown to activate ECs; stabilize tumor vessels; and enhance anti-tumor immunity in pancreatic neuroendocrine, colorectal, and melanoma tumor models (Calcinotto et al., 2012; Johansson et al., 2012; Lu et al., 2015; Shen et al., 2016). Thus, there may be synergy between IFN-γ and TNF-α in promoting vessel normalization. NK cells, macrophages, and T cells were all induced to express these cytokines in the tumor following AIP treatment. However, the innate cells serve as critical sources of these cytokines because depletion of macrophages or NK cells followed by adoptive transfer of *Ifng*^−/−^ innate cells led to a failure of the combination therapy. Notably, ICB has been shown to normalize tumor vessels in models of breast cancer (Tian et al., 2017). We also observed increased pericyte coverage and ameliorated hypoxia in ICB-treated B16F10 tumors, suggesting that succeeding ICB doses may continue the vessel normalization benefit initiated by 1X AIP.

Combinations of checkpoint blockade with antibodies such as trastuzumab and cetuximab, or with long half-life IL-2, have been safely tested in patients with advanced HER2-positive gastric cancer (Chung et al., 2021), recurrent/metastatic head and neck cancer (Sacco et al., 2020), and other advanced solid tumors (Diab et al., 2020). With these precedents, determination of the dosing levels and schedules for clinical translation of the 1X AIP therapy appears feasible. Although the 2.5F-Fc antibody surrogate used here has not been tested in patients yet, its ability to bind integrins overexpressed in multiple human tumors makes it a promising candidate for clinical testing (Van Agthoven et al., 2019; Moore et al., 2013; Sheldrake and Patterson, 2014).

### Limitations of the study

There are some limitations of the study. Our mechanistic studies primarily focused on transplanted tumor models, which do not fully capture characteristics of the human TME. In addition, the organ environment has been shown to impact responses to anti-tumor immunotherapies (Lehmann et al., 2017). Thus, investigations using orthotopic or GEMMs in organs other than the skin are needed to evaluate the roles of NK cells and macrophages in antibody-dependent immunotherapies for tumors at other tissue sites. Similarly, assessment in metastatic tumor models would allow a more comprehensive comparison of these cells’ function in primary versus distant tumor sites. Lastly, since humans and mice may have different tolerance to AIP priming or subsequent combination of PD-1 and CTLA-4 blockade, translation of the proposed priming strategy to clinical testing would require careful monitoring for potential toxicities associated with the combination therapy.

In summary, we have demonstrated a three-component priming strategy that, when combined with ICB treatment, elicits a strong endogenous immune response capable of curing a majority of mice bearing large, initially ICB-insensitive tumors. The success of this regimen provides preclinical rationale for combining approved checkpoint inhibitors with tumor-targeting antibodies and cytokines that synergize innate and adaptive immunity to enhance anti-tumor efficacy.

## STAR★METHODS

### RESOURCE AVAILABILITY

#### Lead contact

Further information and requests for resources and reagents should be directed to and will be fulfilled by the lead contact, Darrell Irvine (djirvine@mit.edu).

#### Materials availability

This study did not generate new unique reagents.

#### Data and code availability

- RNA-seq data have been deposited at GEO: GSE184599 and are publicly available as of the date of publication.
- This paper does not report original code.
- Any additional information required to reanalyze the data reported in this paper is available from the lead contact upon request.

### EXPERIMENTAL MODEL AND SUBJECT DETAILS

#### Animals

Wild-type C57BL/6J mice, *Batf3*^−/−^ mice (B6.129S(C)-*Batf3*^tm1Kmm^/J), *Fcgr3*^−/−^ mice (B6.129P2-*Fcgr3*^tm1Jsv^/2J), Pmel-1/Thy1.1 mice (B6.Cg-*Thy1*^a^/Cy Tg(TcraTcrb)8Rest/J), *Ifng*^−/−^ mice (B6.129S7-*Ifng*^tm1Ts^/J) and IFN-γ reporter mice (B6.129S4-*Ifng*^*tm3.1Lky*^/J) were purchased from the Jackson Laboratory. Tyr:Cre-ER^+^, LSL-*Braf*^*V600E*^, *Pten*^*fl/fl*^, R26-LSL-SIY mice (designated BP-SIY) were generated by S. Spranger (MIT) and T. F. Gajewski (University of Chicago) from mouse strains obtained from L. Chin, M. MacMahon, and T. Mak. Mice used in studies were females at 8–10 weeks of age, except that BP-SIY mice were used at 6–14 weeks and both male and female mice were used. They were housed on a 12 hr light/dark cycle at 23°C with food and water *ad libitum* and were randomly assigned to experimental groups. All animal work was conducted under the approval of the Massachusetts Institute of Technology (MIT) Division of Comparative Medicine in accordance with federal, state and local guidelines.

#### Cell lines

B16F10 cells were purchased from American Type Culture Collection (ATCC). TC-1 cells were kindly provided by T.C. Wu (Johns Hopkins University). YUMM1.7 cells were kindly provided by M.W. Bosenberg (Yale University). KP cells (derived from lung tumors of *Kras*^LSL-G12D/+^;*Trp53*^flox/flox^ (KP) mice) and B16F10-ZsGreen cells were kindly provided by T. Jacks and R. Hynes, respectively (MIT). Expi293 cells were purchased from Life Technologies. Sex of all the cell lines used in the study is unspecified due to lack of public record.

B16F10, B16F10-ZsGreen and KP cells were cultured in complete Dulbecco’s modified Eagle’s medium (DMEM, GE Healthcare Life Sciences; supplemented with 10% FBS, 100 units/ml penicillin, 100 μg/ml streptomycin, and 4 mM l-alanyl-lglutamine), while TC-1 cells were cultured in complete RPMI medium (GE Healthcare Life Sciences) and YUMM1.7 cells were cultured in complete DMEM/F12 with GlutaMAX medium (GIBCO) supplemented with 1X minimal essential medium (MEM) non-essential amino acids (NEAA, GE Healthcare Life Sciences). T cells and splenocytes were cultured in RPMI with 10% heat-inactivated FBS, 20 mM HEPES, 1 mM sodium pyruvate, 0.05 mM β-mercaptoethanol, 100 units/ml penicillin, 100 μg/ml streptomycin, 2 mM L-alanyl-L-glutamine, and 1X MEM NEAA. Expi293 cells were cultured in FreeStyle medium (Life Technologies). All cell lines and assay cultures were maintained at 37°C and 5% CO_2_. All cells were tested regularly for mycoplasma contamination and for rodent pathogens, and none used tested positive at any point.

### METHOD DETAILS

#### Cloning, protein and amphiphile vaccine production

2.5F-Fc was generated by fusing an engineered integrin-targeting cysteine knot peptide with a mouse IgG2c Fc domain and was synthesized as previously described (Kwan et al., 2017). Briefly, DNA sequences encoding the fusion protein was synthesized as genomic blocks (Integrated DNA Technologies) and cloned into gWIZ expression plasmids (Genlantis). A D265A mutation or L234A, L235A and P329G mutations found in mouse IgG2a were introduced to corresponding positions in the IgG2c Fc domain to generate two types of effector attenuating 2.5F-Fc. Similarly, the coding sequences of mouse serum albumin fused to murine IL-2 (MSA-IL-2) with a G_3_S linker and a C-terminal 6X His tag, as well as the heavy and light chains of mouse TA99 antibody, were constructed into the gWIZ vector as described previously (Moynihan et al., 2016; Zhu et al., 2015).

Plasmids were amplified in 5-alpha Competent *E. coli* (New England Biolabs) and purified using ZymoPURE Plasmid Miniprep Kit (Zymo Research). They were then transiently transfected into Expi293 cells using the ExpiFectamine 293 Transfection Kit (Thermo Fisher Scientific). Cell culture supernatants were collected 6 days post-transfection. Antibodies (e.g., TA99 and 2.5F-Fc) and MSA-IL-2 were purified in an ÄKTA pure chromatography system using HiTrap Protein A affinity columns and HisTrap HP His tag protein purification columns, respectively (GE Healthcare Life Sciences). All in-house produced proteins were ensured to contain minimal levels of endotoxin (< 0.05 total EU per μg protein) using the Endosafe nexgen-PTS device (Charles River Laboratories).

Amphiphile-CpG (amph-CpG) and -peptide (amph-peptide) were produced as previously described (Liu et al., 2014; Moynihan et al., 2016). Conjugation of an 18-carbon diacyl tail with a G2 spaced class B CpG 1826 was done through solid-phase synthesis (Oligo Factory). Amph-peptides were produced by conjugation of the N-terminal cysteine of the peptide antigens (e.g., TRP2_180–188_: CSVYDFFVWL) to 1,2-distearoyl-sn-glycero-3-phosphoethanolamine-N-[maleimide (polyethylene glycol)-2000] (Laysan Bio).

#### Tumor model establishment and treatment

For B16F10, TC-1 and YUMM1.7 tumor models, 10^6^ tumor cells were injected subcutaneously into the right flank of mice in 100 μL sterile PBS. For the KP tumor model, 5×10^5^ tumor cells were injected intratracheally into the lung of mice in 20 μL sterile PBS followed by a washing step with another 20 μL sterile PBS. Eight days later, the treatment was initiated as described (Figures 1C and 1G) and tumor size was measured as area with endpoint criteria of 200 mm^2^. For the induction of autochthonous melanoma, BP-SIY mice were shaved on the right flank and 5 μL of 4-hydroxytamoxifen (Sigma) at a concentration of 5 mg/mL in ethanol: isopropanol (95:5) were applied on the right flank on day 0. On day 25, the 1X AIP treatment was initiated as in Figures 1C and 1G and tumor size was measured as volume with endpoint criteria of 1,200 mm^3^. For the 1X AIP treatment, the tumor targeting antibody (A) was administered intraperitoneally at 100 μg per dose for TA99 and 400 μg per dose for 2.5F-Fc. As previously described (Moynihan et al., 2016), MSA-IL-2 (I) was administered at 30 μg per dose (equivalent to 6 μg IL-2), anti-PD-1 (P) (clone 29F.1A12, BioXcell) and anti-CTLA-4 (clone 9D9, BioXcell) were injected at 200 μg per dose intraperitoneally. The vaccine (1.24 nmol amph-CpG and 20 μg amph-peptide) was administered subcutaneously at the tail base of the mice. Mice were randomized into different groups right before the first treatment. Tumor area (longest dimension × perpendicular dimension) was used as a proxy of the growth of subcutaneous (s.c.) tumors every 2–3 days, whereas microCT was used to monitor the growth of lung tumors every week. Mouse body weight was also monitored when measuring tumor size. Mice were euthanized when the area of s.c. tumors exceeded 200 mm^2^ or their body condition was below body condition scoring 2 (BCS2).

All cured mice were re-challenged with 10^5^ tumor cells by s.c. injection on the opposite flank (for s.c. tumors) or by intratracheal injection into the lung (for orthotopic lung tumors).

#### Tissue sample dissociation

Tumors, spleens and lymph nodes (LNs) were carefully dissected from tumor-bearing mice. Tumors were weighted. Both inguinal and axillary LNs were taken as tumor draining (on the tumor side) or non-tumor draining (on the opposite side). Red blood cells in spleen and peripheral blood samples were lysed using GIBCO ACK Lysing Buffer (Thermo Fisher). For immunophenotyping analysis or adoptive cell transfer experiments, single cell suspensions were prepared by mechanically dissociating tumors, spleens or LNs against 70 μm filters using the plunger of a 1 mL or 3 mL syringe. Cells were then pelleted and used for downstream applications

For analyses involving endothelial cells, tumor and LNs were processed based on previous reports (Fankhauser et al., 2017; Fletcher et al., 2011). Basically, tumors were cut into small pieces (~2–4 mm^3^) and incubated for 30 min with constant agitation at 37°C in 1 mL of digestion buffer (1 mg/ml collagenase IV and 40 μg/ml DNase I in RPMI-1640). After incubation, cells were centrifuged at 0.5 RCF for 10 s and 950 μL of solution was taken out and quenched in the STOP buffer (PBS containing 2% FBS and 5 mM EDTA). Cell pellets were resuspended and incubated for 45 min with constant agitation at 37°C in 1 mL of another digestion buffer (3.3 mg/ml collagenase D and 40 μg/ml DNase I in RPMI-1640). After incubation, the solution was quenched by adding the STOP buffer. Residual tumor was gently smashed against and filtered through a 70 μm filter to get single cell suspension. LNs were pierced and torn with sharp forceps and incubated for 20 min with constant agitation at 37°C in 1 mL of digestion buffer (0.8 U/ml dispase, 0.2 mg/ml collagenase P and 0.1 μg/ml DNase I in RPMI-1640). Cells were briefly centrifuged and resuspended in 1 mL of digestion buffer for another round of 10 min incubation 37°C. After digestion, solution was quenched with the STOP buffer and filtered through 70 μm filters before staining for flow cytometry.

For RNA sequencing experiments, tumor samples were collected immediately after surgery and submerged in tissue storage solution (Miltenyi Biotec) and were dissociated using the mouse tumor dissociation kit (Miltenyi Biotec). Briefly, tumors were cut into small pieces (~2–4 mm^3^) and transferred to a gentle MACS C tube containing 2.35 mL RPMI 1640, 100 μL Enzyme D, 50 μL Enzyme R and 12.5 μL Enzyme A. The program 37C_m_TDK_1 was used on a gentle MACS Octo Dissociator with Heaters to fully dissociate tumor tissue. After digestion, the solution was diluted in 10 mL RPMI 1640 and filtered through a prewetted 70 μm filter. Cells were pelleted and used for positive selection of immune cells using mouse CD45 MicroBeads (Miltenyi Biotec).

#### Flow cytometry

Processed cells were incubated with TruStain FcX (anti-mouse CD16/32) antibody (Biolegend) and then stained with antibodies in 1% BSA and 5mM EDTA in PBS for 20 min at 4 °C. Viability was assessed by staining with fixable Live/Dead Aqua (Thermo Fisher). Annexin V and PI staining was performed using an FITC Annexin V Apoptosis Detection Kit I (BD) according to manufacturer’s instructions. Intracellular cytokine staining (ICS) was performed as described previously (Moynihan et al., 2016). Peptides used for restimulation were 10 μg/ml of the relevant antigen: TRP2180–188 (SVYDFFVWL). In cases where no vaccine was used for treatment, intratumoral immune cells were co-incubated with tumor cells at 37 °C for 4 hours in the presence of Brefeldin A after mechanical dissociation. Cells were then stained with fluorophore-conjugated antibodies against surface markers and intracellular cytokines using the BD Cytofix/Cytoperm kit (BD). Staining of nuclear proteins (e.g., FOXP3) was done with the Foxp3 / Transcription Factor Staining Buffer Set (eBioscience). Flow cytometry was performed on a BD Fortessa instrument. Analysis of flow cytometry data was done using FlowJo (Treestar) software.

#### FTY720 treatment

Tumor-bearing mice were treated with 1.5mg/kg FTY720 (or vehicle control) through intraperitoneal injections daily, starting 2 days before 1X AIP treatment. Three days later, the number of immune cells in the blood and tumors were analyzed using flow cytometry.

#### Tumor cytokine Luminex assay and ELISA

Tumors were harvested at day 11 (3 d after first treatment). All tumors from a treatment group were collected, up to a maximum of ~150 mg, for processing in a bead beater tube. Tissue samples were put into PBS (5 μl/mg tumor) supplemented with protease inhibitor cocktail (Roche) according to manufacturer’s instructions. Samples were mechanically disassociated on a bead beater and supernatants were transferred into spin filters after 5min centrifugation at highest speed. Filtered lysates were diluted 1:4 with PBS and were sent for Luminex analyses (Eve Technology). Cytokines, chemokines and growth factors concentrations were normalized to total protein input and levels were clustered by Cluster 3.0 developed by M. Eisen at Stanford University. Values below the limit of detection were set to 0. Levels of FLT3L and XCL1 in tissue culture media were determined by ELISA kits according to manufacturer’s instructions (R&D systems).

#### Enzyme-linked immunosorbent assay

Target B16F10 and B16-TRP2-KO cells were treated with 500 U/ml mIFN-γ (Peprotech) for 12 h, then irradiated (120 Gy). Effector cells were splenocytes isolated from untreated mice or mice that had been treated with various treatments. A mouse IFN-γ ELISPOT Kit (BD) was used. Targets cells were seeded at 25,000 cells per well. Effector cells were seeded at 10^6^ cells per well. TRP2_180–188_ was added to effector cells at 10 μg/ml in peptide stimulation conditions. Plates were wrapped in foil and cultured for 24 h, then developed according to manufacturer’s protocol. Plates were scanned using a CTL-ImmunoSpot Plate Reader, and data were analyzed using CTL ImmunoSpot Software.

#### Antibody-mediated depletions and blocking

Cellular subsets and cytokines were depleted by intraperitoneally administering depleting antibodies (BioXCell) beginning 1 d before therapy as we previously reported (Moynihan et al., 2016): CD8 T cells with anti-CD8-α (clone 2.43, 400 μg every 3 days), CD4 T cells with anti-CD4 (clone GK1.5, 400 μg every 3 days), NK cells with anti-NK1.1 (clone PK136, 400 μg every 3 days), neutrophils with anti-Gr-1 (clone RB6–8C5, 400 μg every 2 days), macrophages with anti-F4/80 (clone CI:A3–1, 200 μg every day) (Lin et al., 2017), IFN-γ with anti- IFN-γ (clone XT3.11, 200 μg every 3 days), TNF-α with anti-TNF-α (clone XMG1.2, 500 μg every 2 days) and CXCL9 with anti-CXCL9 (clone MIG-2F5.5, 300 μg every 2 days). VEGFR2 was blocked by anti-VEGFR2 (clone DC101, 500 μg every 3 days). Apoptosis of intratumoral T cells were induced with anti-CD3ε F(ab’)2 (clone 145–2C11, 50 μg for intratumoral injection or 100 μg for systematic i.p. injection every day) (Besançon et al., 2017) to avoid toxicity associated with full anti-CD3 antibodies in treated mice (data not shown). Cellular depletions of CD3^+^ T cells, CD8^+^ T cells, CD4^+^ T cells, neutrophils, macrophages and NK cells were confirmed by flow cytometry of PBMCs (Figures S3A and S4A).

#### Adoptive cell transfer

Thy1.1+ pmel T cells were harvested from the spleens of Pmel transgenic mice and negatively selected for CD8 with a magnetic bead enrichment method (STEMCELL Technologies). Cells were labeled with 5 μM of CSFE (Thermo Fisher) and washed 3 times with PBS and injected i.v. at 1 × 10^5^ cells per mouse.

For adoptive transfer of NK cells and macrophages, endogenous cells were depleted with neutralizing antibodies against NK cells and macrophages in B16F10-bearing *Fcgr3*-KO or WT mice 1 day before the treatment. Syngeneic WT CD45.1+ or *Ifng*-KO NK cells and macrophages were harvested and selected using a magnetic bead enrichment method (Miltenyi Biotec) and *Ifng*-KO cells were labeled with 5 μM CellTrace Violet or CFSE (Thermo Fisher). NK cells and macrophages were then i.v. injected at 1 × 10^6^ cells (Tai et al., 2013) and 3–4 × 10^6^ cells (Hagemann et al., 2008) per mouse, respectively.

#### Liver enzyme measurements

Serum was isolated from B16F10-bearing mice 3d after 1x AIP treatment. AST and ALT levels were quantified by using a colorimetric aspartate aminotransferase activity assay kit (Sigma Aldrich) or alanine aminotransferase activity assay kit (Sigma Aldrich), respectively, according to the manufacturer’s protocol.

#### Immunofluorescence, imaging and processing

Following various treatments, tumor samples were harvested and incubated overnight on swivel shaker in 50 mM phosphate buffer with 1% paraformaldehyde, 100 mM L-lysine buffer (pH 7.4) and 0.2% sodium periodate. Samples were then washed in PBS for 10 min at room temperature three times and were perfused and cryoprotected with 30% sucrose in PBS on a swivel shaker at 4°C overnight. Then samples were then quickly rinsed in PBS, dried on paper towel, embedded in OCT in and frozen on dry ice before 8–10-μm sectioning (KI Histology Facility). Sections were dried at room temperature for at least 60 min, then rehydrated in PBS for 5 min. They were permeabilized and blocked in rat Immunomix (10% rat serum, 0.05% sodium azide, 0.3% Triton X-100 and 0.2% BSA in PBS for 30 min), followed by overnight staining with primary antibodies anti-CD31-AF647 (clone MEC13.3, Biolegend) and anti-NG2-AF488 (polyclonal, Sigma-Aldrich). Slides were washed in PBS with 0.1% Tween-20 for 5 min three times, washed in PBS for 5 min once, fixed in PBS with 1% paraformaldehyde for 2 min, washed twice in PBS for 5 min and covered by VECTASHIELD Antifade Mounting Medium with DAPI (Vector Labs). The stained slides were imaged using a Leica SP8 confocal microscope and the images were processed using ImageJ (National Institutes of Health).

For tumor vessel patency and permeability assessment, B16F10-bearing mice were left untreated or treated with AIP or ICB for 3 days before being intravenously injected with 70 kD dextran (Thermo Fisher) and a DNA-staining dye Hoechst (Thermo Fisher) 5–10 min before systematic perfusion with PBS (Huang et al., 2012; Tian et al., 2017). Tumors were harvested and fixed with 4% paraformaldehyde overnight, washed and embedded in a 3 wt% low melting point agarose at 37 °C then allowed to cool and solidify on ice for 15 min. The 100-μm sections were prepared using a Vibratome (Leica VT1000S) and suspended in ice-cold PBS then transferred into a blocking solution containing 10% rat serum, 0.2% Triton X-100 and 0.05% sodium azide overnight at 37 °C before immunostaining. Histology sections were stained for CD31 (clone MEC13.3, Biolegend) at 1:100 dilution in blocking buffer overnight at 37 °C, followed by washes with PBS 0.05% Tween and mounted on a glass slide with ProLong Diamond antifade mounting medium (Life Technologies). The stained slides were imaged using a TissueFAXS Whole Slide Scanning System (TissueGnostics GmbH) and analyzed using TissueQuest 7.0 (TissueGnostics GmbH).

For tumor hypoxia level determination, B16F10-bearing mice were untreated or treated with AIP or ICB for 3 days before intravenously injected with a hypoxia-detecting agent EF5 3 hr before euthanasia according to manufacturer’s instructions (Sigma Aldrich). Mice without EF5 injections were used as negative controls. DyLight 649 labeled lectin (Vector Labs) and Hoechst 33342 (Thermo Fisher) were i.v. injected 5–10 min before euthanasia to label vasculatures and cell nuclei. Tumors were collected and cryo-sectioned as described above. Sections were fixed and stained with AF488 conjugated antibodies ELK3–51 against EF5 adducts according to manufacturer’s instructions (Sigma Aldrich). The stained slides were imaged using a TissueFAXS Whole Slide Scanning System (TissueGnostics GmbH) and EF5^+^ areas were quantified using TissueQuest 7.0 (TissueGnostics GmbH).

#### Whole-tumor clarification and light sheet microscopy

B16F10-bearing mice were left untreated or treated with AIP or ICB for 3 days before being intravenously injected with 100 μg anti-CD31-AF647 (clone MEC13.3, Biolegend) 5–10 min before euthanasia. Tumors were harvested and fixed in 4% paraformaldehyde at 37 °C for 24 hr with agitation. They were then clarified via a modified CUBIC method (Kubota et al., 2017). Samples were first delipidated in 20% methanol in PBS for 30 min. A series of stepwise increases in methanol percentage (40%, 60%, 80% and 100%) followed, each step for 30 min. They were then bleached in freshly made 3: 2: 1 MeOH: H_2_O_2_: DMSO solution at 37 °C overnight or longer until tumors turned pale. They were then rehydrated with the following series of methanol-PBS solutions for 30 min each: 100%, 80%, 60%, 40%, 20%, 0%. Next, tumors were washed with water 3 times and placed into 10 mL of a 1:1 mixture of CUBIC-R index-matching solution (45 wt% antipyrine and 30 wt% nicotinamide in water) at 37 °C for 2 days, followed by 10 mL of undiluted CUBIC-R for 2 days or as long as needed for adequate clarification. Clarified tumors were imaged in CUBIC-R solution using a LaVision Ultramicroscope II Light Sheet Microscope at × 1.25 optical zoom using the 640-nm laser at 100 ms exposure time on an Andor Neo camera. Snapshots were generated in the FIJI package of ImageJ. The sizes of tumor vessels were analyzed in MATLAB R2018a (MathWorks).

#### qPCR analysis

Tumor infiltrating NK cells and macrophages were isolated as described above. Total RNA was extracted and reverse transcribed using the RNeasy Micro Kit (QIAGEN) and High-Capacity RNA-to-cDNA Kit (Thermo Fisher), followed by amplification with TaqMan Universal Master Mix (Thermo Fisher) and specific primers for *Ifng*, *Tnf*, *Gzmb*, *Cxcl9*, *Cxcl10*, *Xcl1*, *H2-Aa*, *Nos2* and *Actb* from Thermo Fisher Scientific and detected by a Roche LightCycler 480. The Ct values were normalized with the housekeeping gene mouse *Actb* for comparison.

#### RNA sequencing, mapping and analysis

A total of 3,000–20,000 tumor infiltrating NK cells and macrophages were sorted by a FACSAria III into RLT buffer with β-mercaptoethanol (QIAGEN). RNA extraction was performed using RNeasy micro kits (QIAGEN). RNaseq reads were used to quantify gene expression with Salmon (version 1.1.0, (Patro et al., 2017)) using a target transcriptome derived from the mm10 primary assembly and an ensembl v.98 annotation and the mm10 genome as a selective alignment decoy. The resulting counts and transcript per million (TPM) values were assembled using tximport (version 1.12.3, (Soneson et al., 2015)). The TPM values were transformed to log2 space with a plus 1 offset. Differential expression analysis was done using integer count data and DESeq2 (version 1.24.0, (Anders and Huber, 2010; Love et al., 2014)) with apeglm log fold change shrinkage (Zhu et al., 2019). Significant genes were defined as those having an absolute log2 fold change > 1 and an adjusted p value < 0.05. Pre-ranked Gene Set Enrichment Analysis (version 4.0.3, (Subramanian et al., 2005)) was run using the DESeq2 Wald statistic as a ranking metric and gene set collections from msigDB (version 7.0, (Liberzon et al., 2015)). GSEA enrichment plots and dot plots were prepared with R version ggplot2 (version 3.2.1; (Wick-ham, 2016)). NK cell- and macrophage-specific genes related to their activation status and subtypes shown in Figures 4C and 4D were curated based on previous reports (Bezman et al., 2012; Orecchioni et al., 2019).

### QUANTIFICATION AND STATISTICAL ANALYSIS

Statistics were analyzed using GraphPad Prism software. All graphs represent mean and standard deviations unless otherwise noted. Comparisons of more than two groups were performed using a one-way or two-way ANOVA with a Tukey or Dunnett post hoc test to determine statistical significance. For comparisons of experiments with only two groups, two-tailed unpaired Student’s t tests were used. Survival curves were analyzed by using the log-rank (Mantel–Cox) test. p values < 0.05 were considered statistically significant. All of the statistical details of experiments can be found in the figure legends. Investigators were not blinded to group assignment during experimental procedures or analysis.

## Supplementary Material

1

## Figures and Tables

**Table:** KEY RESOURCES TABLE

REAGENT or RESOURCE	SOURCE	IDENTIFIER
Antibodies		
Anti-mouse CD3 (17A2) PE-Cy7	BioLegend	100220; RRID:AB_1732057
Anti-mouse CD8a (53-6.7) BUV395	BD Biosciences	563786; RRID:AB_2732919
Anti-mouse CD8a (53-6.7) BV421	BioLegend	100738; RRID:AB_11204079
Anti-mouse CD4 (RM4-5) FITC	BioLegend	100510; RRID:AB_312713
Anti-mouse CD25 (PC61) APC-Cy7	BioLegend	102026; RRID:AB_830745
Anti-mouse CD137 (17B5) APC	BioLegend	106110; RRID:AB_2564297
Anti-mouse PD-1 (29F.1A12) BV421	BioLegend	135218; RRID:AB_2561447
Anti-mouse PD-1 (J43) BV421	BD Biosciences	562584; RRID:AB_2737668
Anti-mouse CTLA-4 (UC10-4B9) BV605	BioLegend	106323; RRID:AB_2566467
Anti-mouse CD152 (UC10-4F10-11) PE-CF594	BD Biosciences	564332; RRID:AB_2732917
Anti-mouse CD161 (PK136) PerCP-Cy5.5	BioLegend	108728; RRID:AB_2132705
Anti-mouse CD49b (DX5) PerCP-Cy5.5	BioLegend	108916; RRID:AB_2129358
Anti-mouse CD69 (H1.2F3) BV785	BioLegend	104543; RRID:AB_2629640
Anti-mouse CD107a (1D4B) BV711	Biolegend	121631; RRID:AB_2783065
Anti-mouse CD45.2 (104) BUV737	BD Biosciences	612778; RRID:AB_2870107
Anti-mouse Ly-6C (HK1.4) PE-Cy7	BioLegend	128018; RRID:AB_1732082
Anti-mouse Ly-6G (1A8) PerCP-Cy5.5	BioLegend	127616; RRID:AB_1877271
Anti-mouse CD11c (N418) FITC	BioLegend	117306; RRID:AB_313775
Anti-mouse CD11b (M1/70) APC-Cy7	BioLegend	101226; RRID:AB_830642
Anti-mouse CD24 (M1/69) BV711	BioLegend	563450; RRID:AB_2738213
Anti-mouse MHC II (M5/114.15.2) BV605	BioLegend	107639; RRID:AB_2565894
Anti-mouse F4/80 (T45-2342) BUV395	BD Biosciences	565614; RRID:AB_2739304
Anti-mouse CD86 (GL-1) PE-Dazzle 594	BioLegend	105042; RRID:AB_2566409
Anti-mouse CCR7 (4B12) PerCP-Cy5.5	BioLegend	120116; RRID:AB_2291144
Anti-mouse CD169 (3D6.112) APC	BioLegend	142418; RRID:AB_2565641
Anti-mouse CD103 (2E7) PE	BioLegend	121406; RRID:AB_1133989
Anti-mouse CD206 (C068C2) PerCP-Cy5.5	BioLegend	141716; RRID:AB_2561992
Anti-mouse Ki67 (16A8) PE-Dazzle 594	BioLegend	652428; RRID:AB_2632696
Anti-mouse CD31 (MEC13.3) AF488	BioLegend	102514; RRID:AB_2161031
Anti-mouse CD31 (MEC13.3) AF647	BioLegend	102516; RRID:AB_2161029
Anti-mouse CD31 (390) BV605	BioLegend	102427; RRID:AB_2563982
Anti-mouse GP38 (8.1.1) APC-Cy7	BioLegend	127418; RRID:AB_2629804
Anti-mouse NG-2 (polyclonal) AF488	Sigma Aldrich	AB5320A4; RRID:AB_11203143
Anti-human/mouse AN2 (NG-2, 1E6.4) PE	Miltenyi Biotec	130-097-455; RRID:AB_2651235
Anti-mouse VEGFR2 (89B3A5) PerCP-Cy5.5	BioLegend	121918; RRID:AB_893646
Anti-mouse PNAd (MECA-79) AF488	eBioscience	53-6036-82; RRID:AB_10804391
Anti-mouse CD106 (VCAM-1, 429) AF647	BioLegend	105712; RRID:AB_493429
Anti-mouse CD102 (ICAM-1, YN1/1.7.4) BV711	BioLegend	116143; RRID:AB_2876429
Anti-mouse CD62E (10E9.6) BV421	BD Biosciences	740027; RRID:AB_2739799
Anti-mouse CD45.1 (A20) APC	BioLegend	110714; RRID:AB_313503
Anti-mouse Thy1.1 (OX-7) BV711	BioLegend	202539; RRID:AB_2562645
Anti-mouse IFN-γ (XMG1.2) PE	BioLegend	505808; RRID:AB_315402
Anti-mouse IFN-γ (XMG1.2) APC	BioLegend	505810; RRID:AB_315404
Anti-mouse TNF-α (MP6-XT22) FITC	BioLegend	506304; RRID:AB_315425
Anti-mouse Granzyme B (QA16A02) APC	BioLegend	372204; RRID:AB_2687028
Anti-mouse CXCL9 (MIG-2F5.5) eFluor660	eBioscience	50-3009-80; RRID:AB_11218694
Anti-mouse PD-1 (29F1A12)	BioXCell	BE0273; RRID:AB_2687796
Anti-mouse CTLA-4 (9D9)	BioXCell	BE0131; RRID:AB_10950184
Anti-mouse CD3ε F(ab’)2 (145-2C11)	BioXCell	BE0001-1FAB; RRID:AB_2687679
Anti-mouse CD3ε (145-2C11)	BioXCell	BE0001-1; RRID:AB_1107634
Anti-mouse CD8α (2.43)	BioXCell	BE0061; RRID:AB_1125541
Anti-mouse CD4 (GK1.5)	BioXCell	BE0003-1; RRID:AB_1107636
Anti-mouse NK1.1 (PK136)	BioXCell	BE0036; RRID:AB_1107737
Anti-mouse Gr-1 (RB6-8C5)	BioXCell	BE0075; RRID:AB_10312146
Anti-mouse F4/80 (CI:A3-1)	BioXCell	BE0206; RRID:AB_10949019
Anti-mouse IFN-γ (XT3.11)	BioXCell	BE0055; RRID:AB_1107694
Anti-mouse TNF-α (XMG1.2)	BioXCell	BE0058; RRID:AB_1107764
Anti-mouse CXCL9 (MIG-2F5.5)	BioXCell	BE0309; RRID:AB_2736989
Anti-mouse VEGFR2 (DC101)	BioXCell	BE0060; RRID:AB_1107766
Bacterial and virus strains		
5-alpha Competent *E. coli*	New England Biolabs	C2987U
Chemicals, peptides and recombinant proteins		
1,2-distearoyl-sn-glycero-3-phosphoethanolamine-N-[maleimide (polyethylene glycol)-2000]	Layson Bio	100220
4-hydroxytamoxifen solution	Sigma Aldrich	SML1666
GolgiPlug Protein Transport Inhibitor (containing Brefeldin A)	BD Biosciences	BDB555029
Cell Stimulation Cocktail	eBioscience	00-4970-93
FTY720	Sigma Aldrich	SML0700
Protease Inhibitor Cocktail	Roche	5892970001
CSVYDFFVWL	Genscript	SC1848
Recombinant murine IFN-γ	Peprotech	315-05
DNase I	Sigma Aldrich	10104159001
Collagenase IV	Worthington	LS004188
Collagenase D	Sigma Aldrich	11088866001
Collagenase P	Sigma Aldrich	11213865001
Dispase	StemCell Technologies, Inc.	7913
Critical commercial assays		
ZymoPURE Plasmid Miniprep Kit	Zymo Research	D4210
ExpiFectamine 293 Transfection Kit	Thermo Fisher	A14524
GIBCO ACK Lysing Buffer	Thermo Fisher	A10492-01
CSFE	Thermo Fisher	C34554
CellTrace Violet	Thermo Fisher	C34557
LIVE/DEAD Fixable Aqua Dead Cell Stain Kit, for 405 nm excitation	Thermo Fisher	L34966
FITC Annexin V Apoptosis Detection Kit	BD Biosciences	556547
Fixation/Permeabilization Solution Kit	BD Biosciences	554714
Foxp3 / Transcription Factor Staining Buffer Set	eBioscience	00-5523-00
MACS tissue storage solution	Miltenyi Biotec	130-100-008
Mouse tumor dissociation kit	Miltenyi Biotec	130-096-730
Mouse CD45 microbeads	Miltenyi Biotec	130-052-301
NK Cell Isolation Kit, mouse	Miltenyi Biotec	130-115-818
Macrophage Isolation Kit (Peritoneum), mouse	Miltenyi Biotec	130-110-434
EasySep Mouse CD8+ T Cell Isolation Kit	StemCell Technologies, Inc.	19853
Mouse IFN-γ ELISPOT Kit	BD Biosciences	551083
VECTASHIELD Antifade Mounting Medium with DAPI	Vector Labs	H-1200-10
Hoechst 33342, Trihydrochloride, Trihydrate, 100mg	Thermo Fisher	H1399
Dextran, Tetramethylrhodamine, 70,000	Thermo Fisher	D1818
DyLight 649 labeled lectin	Vector Labs	DL-1178
EF5 Hypoxia Detection Kit, Alexa Fluor 488	Sigma Aldrich	EF5-30A4
Aspartate aminotransferase activity assay kit	Sigma Aldrich	MAK055-1KT
Alanine aminotransferase activity assay kit	Sigma Aldrich	MAK052-1KT
RNeasy Micro Kit	QIAGEN	74004
High-Capacity RNA-to-cDNA Kit	Thermo Fisher	4388950
TaqMan Universal Master Mix	Thermo Fisher	4440049
Mouse/Rat FLT3L Quantikine ELISA Kit	R&D systems	MFK00
Mouse XCL1/Lymphotactin DuoSet ELISA	R&D systems	DY486
Deposited data		
RNA-seq data	GEO	GEO: GSE184599
Experimental models: Cell lines		
B16F10 cells	ATCC	CRL-6475; RRID:CVCL_0159
TC-1 cells	T. C. Wu Lab at JHU	N/A
YUMM1.7 cells	M. W. Bosenberg Lab at Yale	N/A
KP cells	T. Jacks Lab at MIT	N/A
B16F10-ZsGreen cells	R. Hynes Lab at MIT	N/A
Expi293F cells	Thermo Fisher	A14527; RRID:AB_2761403
Experimental models: Organism/strains		
C57BL/6J mice	Jackson Laboratory	000624; RRID:IMSR_JAX:000624
*Batf3*^−/−^ mice (B6.129S(C)-*Batf3*^tm1Kmm^/J)	Jackson Laboratory	013755; RRID:IMSR_JAX:013755
*Fcgr3*^−/−^ mice (B6.129P2-*Fcgr3*^tm1Jsv^/2J)	Jackson Laboratory	009637; RRID:IMSR_JAX:009637
Pmel-1/Thy1.1 mice (B6. Cg-*Thy1*^a^/Cy Tg(TcraTcrb)8Rest/J)	Jackson Laboratory	005023; RRID:IMSR_JAX:005023
IFN-γ reporter mice (B6.129S4-*Ifng*^*tm3.1Lky*^/J)	Jackson Laboratory	017581; RRID:IMSR_JAX:017581
*Ifng*^−/−^ mice (B6.129S7-*Ifng*^tm1Ts^/J)	Jackson Laboratory	002287; RRID:IMSR_JAX:002287
BP-SIY mice	Spranger Lab at MIT	N/A
Oligonucleotides		
*Il10* primer	Thermo Fisher	Mm01288386_m1
*Ncr1* primer	Thermo Fisher	Mm01337324_g1
*Klra8* primer	Thermo Fisher	Mm01183337_m1
*Xcl1* primer	Thermo Fisher	Mm00434772_m1
*Flt3l* primer	Thermo Fisher	Mm00442801_m1
*Tnf* primer	Thermo Fisher	Mm00443258_m1
*Ifng* primer	Thermo Fisher	Mm01168134_m1
*Nos2* primer	Thermo Fisher	Mm00440502_m1
*H2-Aa* primer	Thermo Fisher	Mm00439211_m1
*Cxcl9* primer	Thermo Fisher	Mm00434946_m1
*Gzmb* primer	Thermo Fisher	Mm00442837_m1
*Stat1* primer	Thermo Fisher	Mm01257286_m1
*Vegfa* primer	Thermo Fisher	Mm00437306_m1
*Actb* primer	Thermo Fisher	Mm02619580_g1
Recombinant DNA		
gWIZ plasmid	Genlantis	P000200

## Highlights

- Single dose of AIP immunotherapy inflames the tumor microenvironment (TME)
- This sensitizes tumors to immune checkpoint blockade
- NK cells and macrophages destruct tumors and recruit cDC1 and lymphocytes to the TME
- NK cells and macrophage facilitate rapid tumor vasculature remodeling
