# Supplementary material for: Reprogramming NK cells and macrophages via combined antibody and cytokine therapy primes tumors for elimination by checkpoint blockade
Source: Cell Rep. Author manuscript; Available in PMC 2021 Dec 8. (PMC8653865; doi:10.1016/j.celrep.2021.110021)
Supplement: 1 [file NIHMS1759310-supplement-1.pdf]

Cell Reports, Volume *37*

Supplemental information

Reprogramming NK cells and macrophages via combined antibody and cytokine therapy primes tumors for elimination by checkpoint blockade

Chensu Wang, Ang Cui, Maurice Bukenya, Aereas Aung, Dikshant Pradhan, Charles A. Whittaker, Yash Agarwal, Ayush Thomas, Simon Liang, Parastoo Amlashi, Heikyung Suh, Stefani Spranger, Nir Hacohen, and Darrell J. Irvine

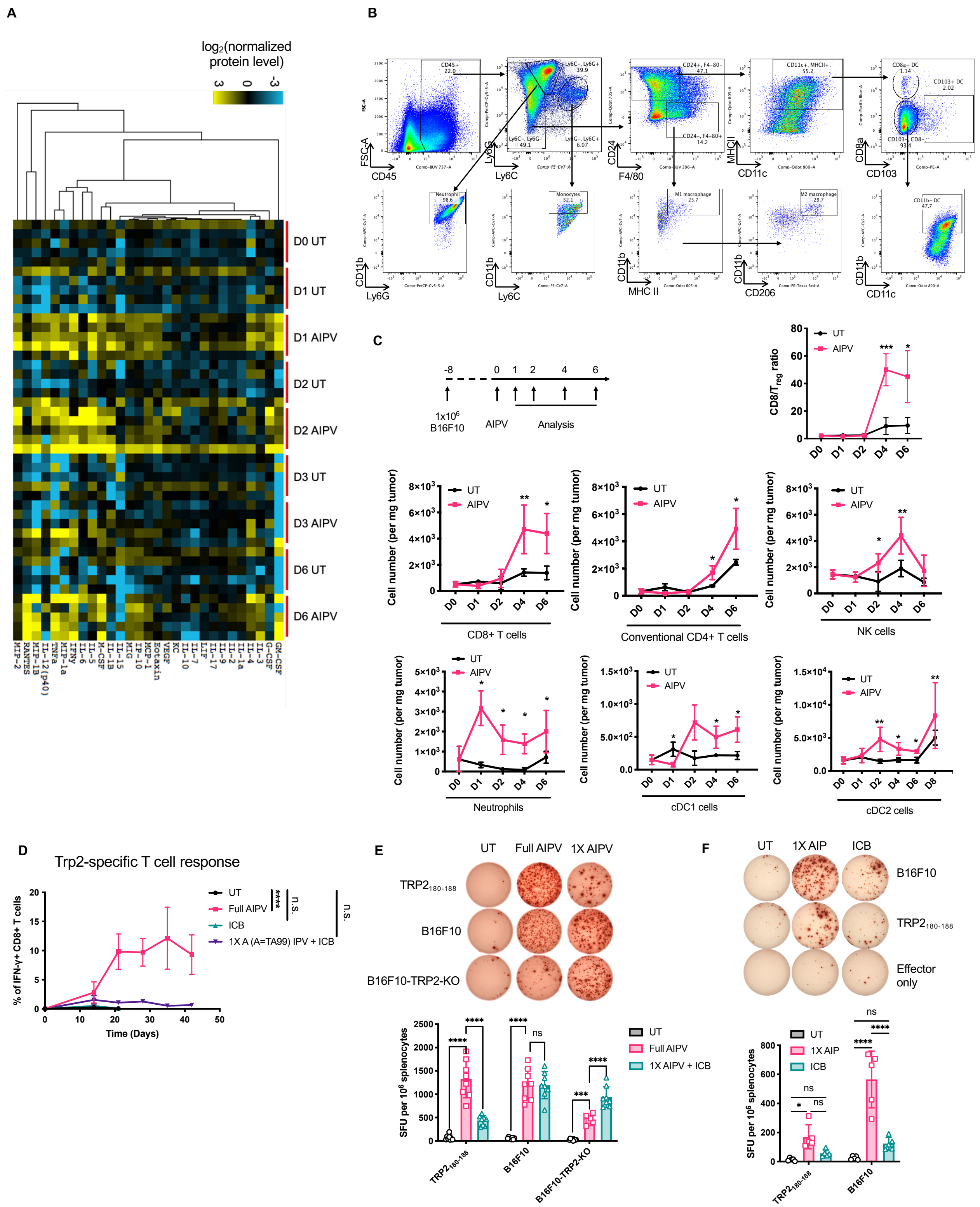

Figure S1, related to Figure 1. Cytokine production, immune infiltration, and T cell responses following a single dose of AIPV or AIP.

(**A**) Mice bearing B16F10 tumors were treated with the AIPV therapy (D0), and tumors were isolated on D0, D1, D2, D3 and D6. Cytokine/chemokine levels were measured by Luminex ELISA

(*n* = 5 C57BL/6 mice/group). Shown is $\log_2$ (normalized protein level/column mean). Row data was clustered based on Euclidean distance.

(**B-C**) Mice with B16F10 tumors were treated as indicated in **Figure 1C**, and tumors were isolated 1, 2, 4, or 6 days post treatment and analyzed by flow cytometry. Shown are gating strategies of indicated intratumoral myeloid cells (after single cell and live cell gating, **B**) and quantification of various immune cells in the tumor (*n*= 5-10 mice per group, **P*<0.05; *****P*<0.0001; versus UT by 2-way ANOVA with the Holm-Šídák test).

(**D**) B16F10-bearing mice were treated with combination therapies as indicated and bled weekly. Blood CD8+ T cell responses to vaccinated TRP2 antigen peptide were assessed by intracellular staining (ICS). Shown were percentages of IFN-γ^+ $CD8^+$ T cells. Statistical comparison was done between UT and treatment groups (*n* = 5 mice per group, *****P*<0.0001; versus UT by 2-way ANOVA with the with the Dunnett test).

(**E**) Groups of B16F10 tumor-bearing mice (n=5 animals/group) received 1X AIPV + ICB treatment or no treatment and splenocytes were isolated at day 16 for IFN-γ ELISPOT. Shown are representative images of ELISPOT plates and quantification of splenocytes cultured with TRP2 antigen peptide, or irradiated TRP2-knockout (KO) B16F10 cells or parental B16F10 cells.

(**F**) Groups of B16F10 tumor-bearing mice (n=5 animals/group) were left untreated or were treated with 1X AIP + ICB or ICB alone, and splenocytes were isolated at day 16 for IFN-γ ELISPOT. Shown are representative images of ELISPOT plates and quantification of splenocytes cultured in the absence of further stimulation (Effector only), TRP2 antigen peptide, or irradiated parental B16F10 cells. SFU, spot-forming unit; **P*<0.05; ****P*<0.001; versus 1X AIP + ICB by two-way ANOVA with the Holm-Šídák test.

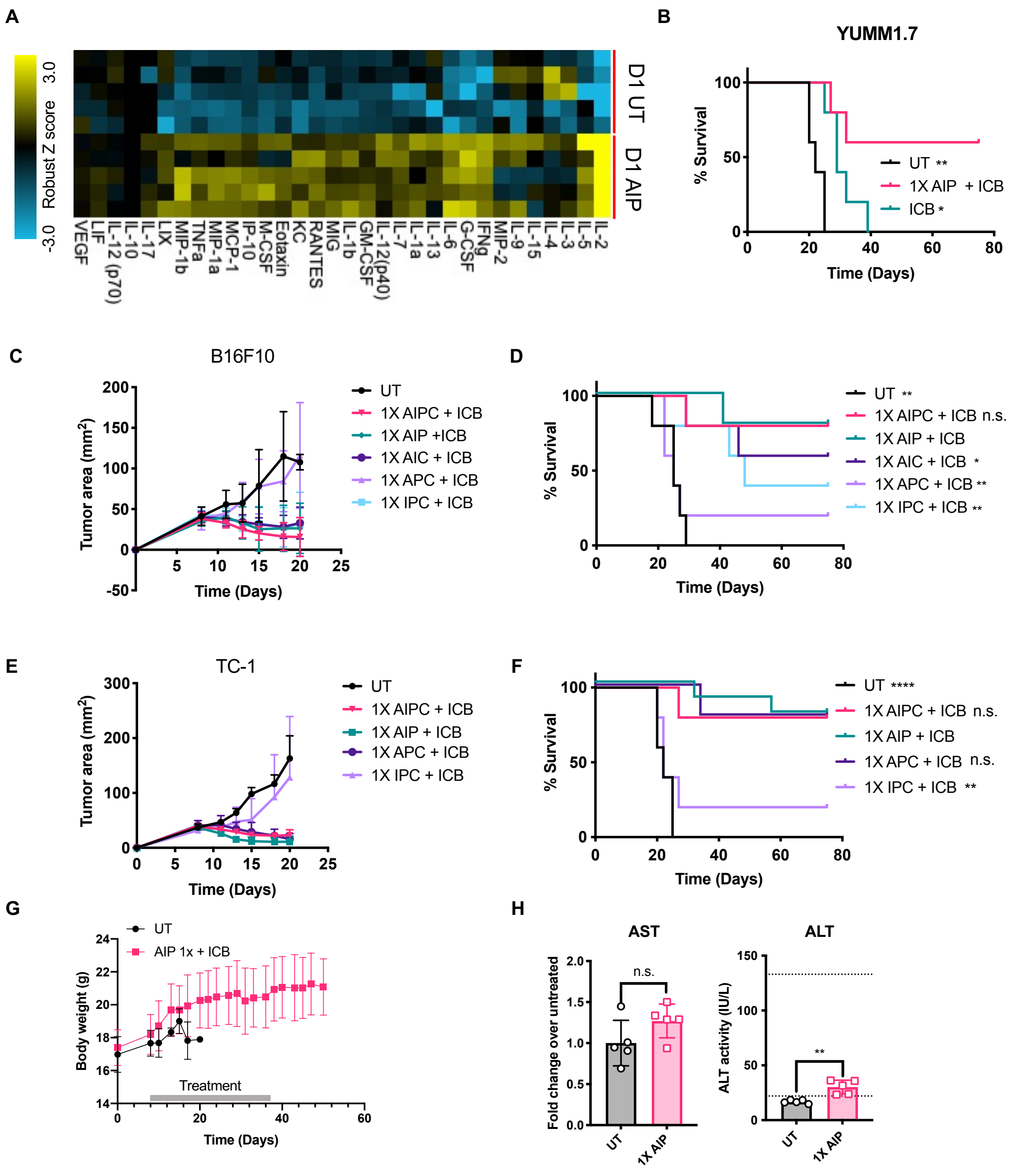

Figure S2, related to Figure 1. 1X AIP treatment is effective in multiple tumor models without overt toxicity.
(**A**) Mice bearing B16F10 tumors were left untreated or treated with AIP. Tumors were isolated on 1-day post treatment. Cytokine/chemokine levels were measured by Luminex ELISA (*n* = 5 mice per group). Shown are heatmaps of the robust Z score.
(**B**) Survival over time for C57BL/6J mice inoculated with 10^6 YUMM1.7 tumor cells s.c. in the flank and treated 8 days later with AIP followed by ICB or ICB alone.
(**C-F**) Average tumor growth (**C, E**) and survival over time (**D, F**) for mice inoculated with 10^6 B16F10 tumor cells (**C, D**) or TC-1 cells (**E, F**) s.c. in the flank and treated 8 days later with AIP, AIPC (AI+αPD-1 and αCTLA-4), AIC (AI+αCTLA-4), APC (A+αPD-1 and αCTLA-4) or IPC (I+αPD-1 and αCTLA-4) once and followed by αPD-1 and αCTLA-4 combination therapy every 3 days for 4 weeks (*n* = 5-10 C57BL/6 mice per group). A = 2.5F-Fc (targeting integrins), I = MSA-IL-2, P = anti-PD-1, C = anti-CTLA-4.
(**G-H**) C57BL/6 mice (n = 5 animals/group) were inoculated with 10^6 B16F10 cells s.c. in the flank and treatment was initiated following the timeline in **Figure 1C** and **1G**. Shown are body weight measurements over time for untreated or 1X AIP + ICB-treated mice (**G**) and analysis of AST and

ALT liver enzyme levels (**H**) in serum of mice 3d post treatment. Dashed lines indicate the normal range of ALT in C56BL/6 mice (22-133 μ/L) (Loeb, et al., 1999).
Throughout, **P*<0.05; ***P*<0.01; ****P*<0.001; *****P*<0.0001; versus 1X AIP + ICB by log-rank test in (**B**, **D**, **F**) and by Welch's t-test with Bonferroni correction in (**H**).

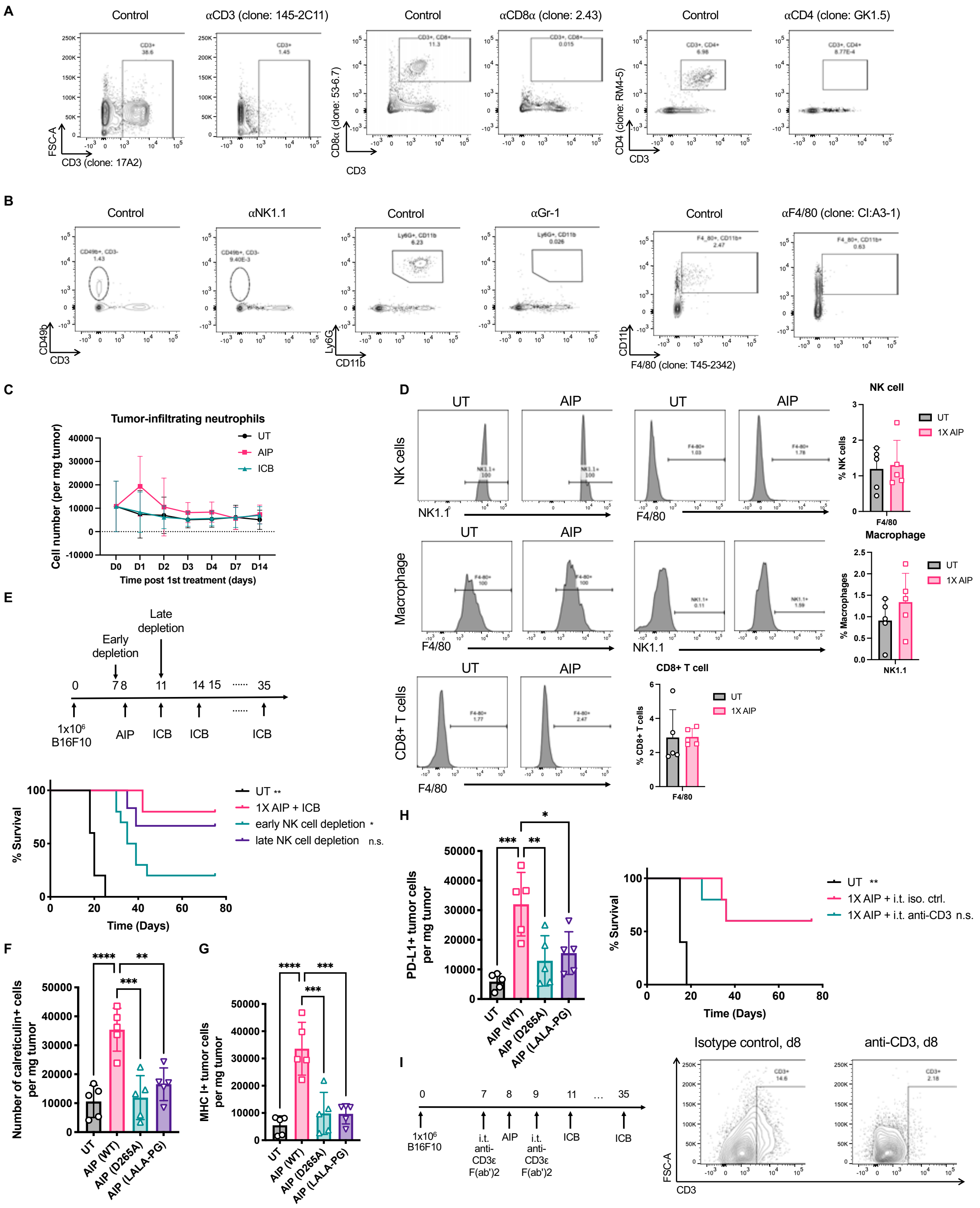

Figure S3, related to Figure 2 and 3. Interaction of 2.5F-Fc antibody surrogate with Fc receptors is critical for therapeutic efficacy of AIP treatment.
(**A-B**) Mice were treated with depleting antibodies against CD3, CD8 and CD4 (**A**) or NK1.1, Gr-1 and F4/80 (**B**). Shown is representative gating for CD3^{+} cells, CD8^{+} T cells and CD4^{+} T cells (**A**)

or NK cells, neutrophils and macrophages (**B**) in blood or spleen 2 days post injection demonstrating effective depletion of each cell type.

(**C**) Mice bearing B16F10 tumors (*n* = 5 animals/group) were left untreated, treated with ICB only, or treated with 1X AIP + ICB as in **Figure 1G**. Tumors were isolated at indicated time points post treatment and were analyzed by flow cytometry. Shown are quantifications of the total number of tumor-infiltrating neutrophils.

(**D**) The expression of F4/80 and NK1.1 on NK cells and macrophages as well as the expression of F4/80 on CD8+ T cells in tumor-bearing mice with or without AIP treatment after 1 day was determined by flow cytometry. Shown are representative histograms and quantifications of the percentage of positive cells.

(**E**) Survival of B16F10-bearing mice over time treated with depleting anti-NK1.1 antibody every 3 days starting 1 day before (early NK cell depletion) or 3 days post (late NK cell depletion) initiation of 1X AIP combination immunotherapy (*n* = 5 C57BL/6 mice per group).

(**F-H**) Quantification of immunogenic (**F**, Calreticulin+) tumor cell death and MHC I (**G**) and PD-L1 (**H**) expression on tumor cells in untreated or AIP-treated tumors using unmodified 2.5F-Fc or D265A- or LALA-PG-mutant 2.5F-Fc as the “A” component.

(**I**) Survival of B16F10-bearing mice over time following intratumoral (i.t.) injection of anti-CD3ε $F(ab')_2$ 1 day before and 1 day post initiation of 1X AIP combination immunotherapy (*n* = 5 C57BL/6 mice per group). Representative flow plots of isotype control vs. anti-CD3ε $F(ab')_2$-treated samples from treated tumors on day 8 demonstrating succcesful depletion of intratumoral T cells.

Throughout, **P*<0.05; ***P*<0.01; ****P*<0.001; versus 1X AIP by log-rank test (**E, I**) and one-way ANOVA with the Holm-Šídák test (**F-H**).

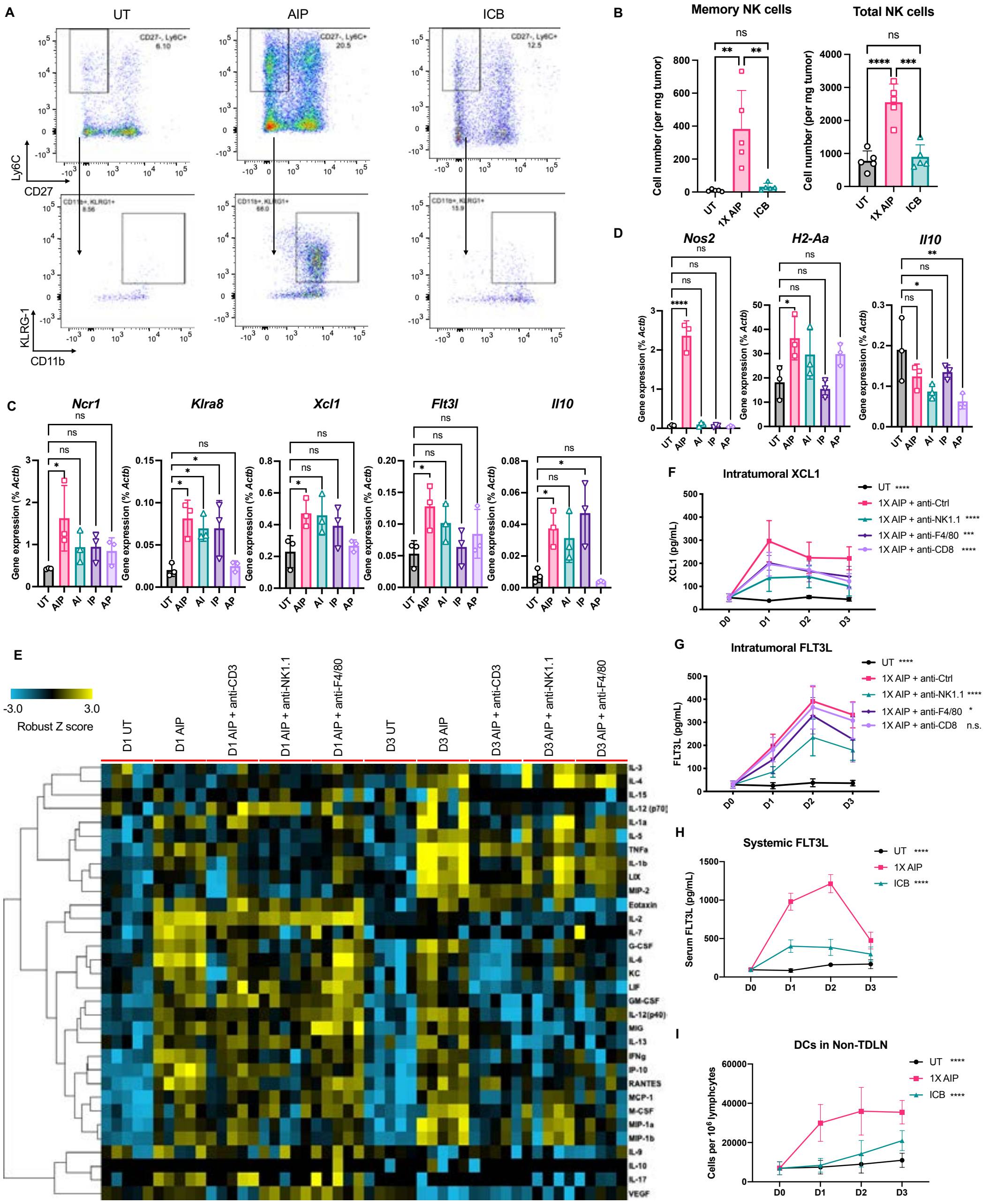

Figure S4, related to Figures 4 and 5. AIP induces a broad range of transcriptional changes in macrophages and NK cells accompanying cytokine and chemokine production in the TME.

(**A-B**) Mice bearing B16F10 tumors (n = 5 animals/group) were left untreated, treated with AIP or ICB. Tumors were isolated 3-days post treatment and were analyzed by flow cytometry. Shown are representative plots (**A**) and quantifications (**B**) of the total number of tumor-infiltrating NK cells (defined as CD45+, CD3-, NK1.1+) and memory NK cells (defined as CD27-, Ly6C+, CD11b+ and KLRG1+ NK cells). (**C-D**) Mice were treated the same as in **Figure 4E-F**. Shown is normalized expression of additional example genes from NK cell (**C**) and macrophage (**D**) samples used in RNA-seq and determined by qPCR.

(**E**) Mice bearing B16F10 tumors were untreated, treated with AIP (D0) in the presence of isotype control antibodies or depleting antibodies against CD3, NK1.1 or F4/80 (administered twice every 3 days starting 1d before AIP treatment. Tumors were isolated on day 1 and day 3 for analysis of cytokine/chemokine levels by Luminex ELISA (n = 5 mice per group). Shown are heatmaps of the robust Z score.
(**F-G**) Intratumoral levels of XCL1 (**F**) and FLT3L (**G**), and the systemic level of FLT3L (**H**) were determined by ELISA in mice in the presence or absence of depleting antibodies for CD8+ T cells (CD8α), NK cells, or macrophages injected 1 day before AIP or ICB treatment (n = 5 C57BL/6 mice per group). Tumor or serum samples were collected on the day of treatment (D0) or 1-3 days post treatment (D1-D3).
(**I**) The number of DCs in the inguinal and axillary lymph nodes of the opposite side of tumor inoculation was determined in mice treated with AIP or ICB by flow cytometry. The sample collection timeline is the same as in (**D-F**).
Throughout, **P*<0.05; ***P*<0.01; ****P*<0.001; *****P*<0.0001; versus 1X AIP by one-way ANOVA with the Dunnett test (**C-D**) and two-way ANOVA with the Dunnett test (**A**-**B** and **F-I**).

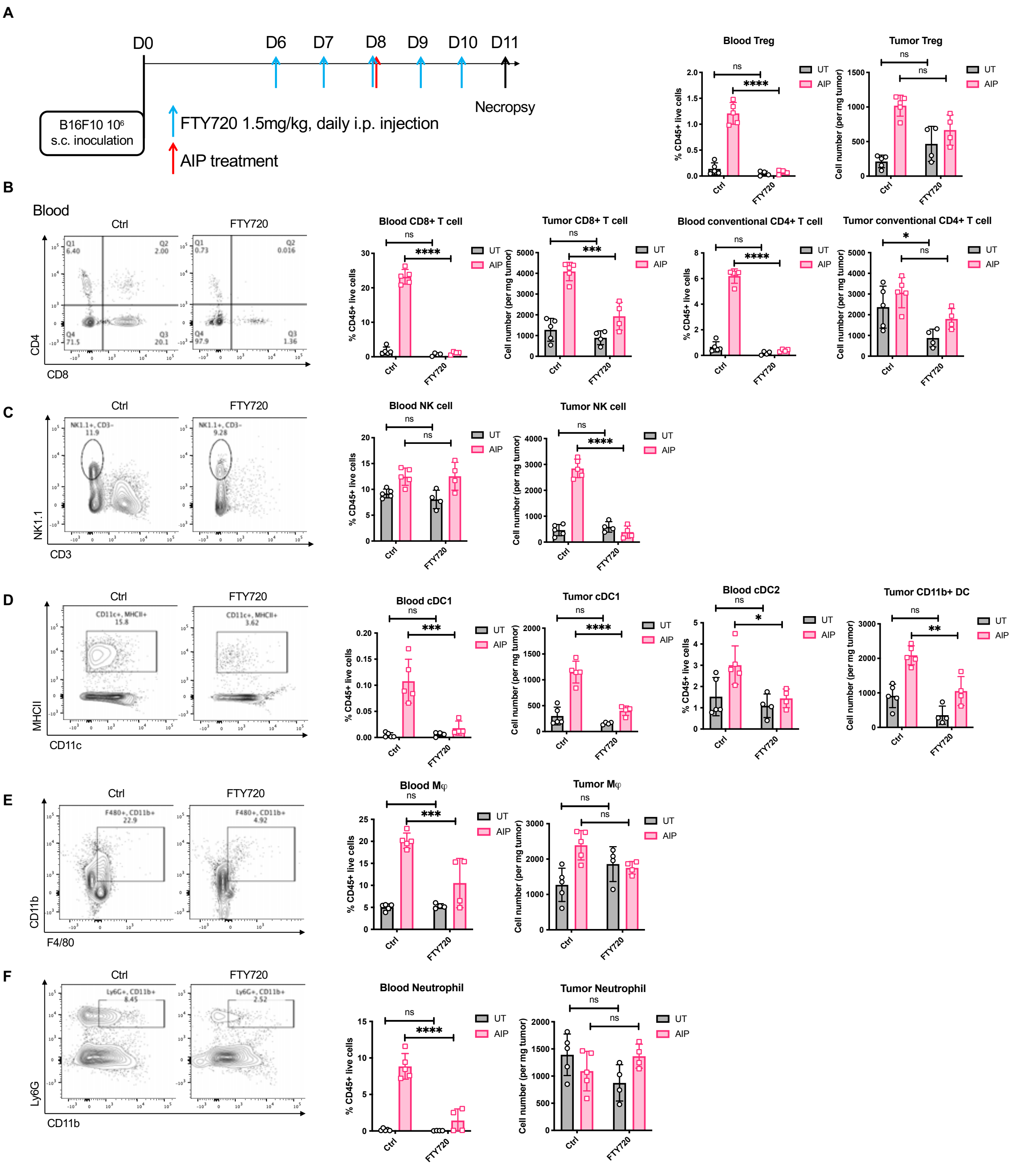

Figure S5, related to Figure 5. Accumulation of immune cells in AIP-treated tumors is inhibited by FTY720 treatment.

(**A**) Schematic of the FTY720 treatment schedule.

(**B-F**) Tumor-bearing mice were treated with 1.5mg/kg FTY720 (or vehicle control) through intraperitoneal injections daily, starting 2 days before 1X AIP treatment. Three days later, the number of T cells (**B,** CD8+ T cells gated as CD3$^+$, CD8α$^+$; <u>conventional CD4$^+$ T cells gated as CD3$^+$, CD4$^+$, FOXP3$^-$;</u> and Tregs gated as CD3$^+$, CD4$^+$, CD25$^+$, FOXP3$^+$), NK cells (**C**), cDC1 and

cDC2 (**D**), macrophages (**E**) and neutrophils (**F**) in the blood and tumors were analyzed using flow cytometry.

Throughout, **P*<0.05; ***P*<0.01; ****P*<0.001; *****P*<0.0001; by two-way ANOVA with the Holm-Šídák test.

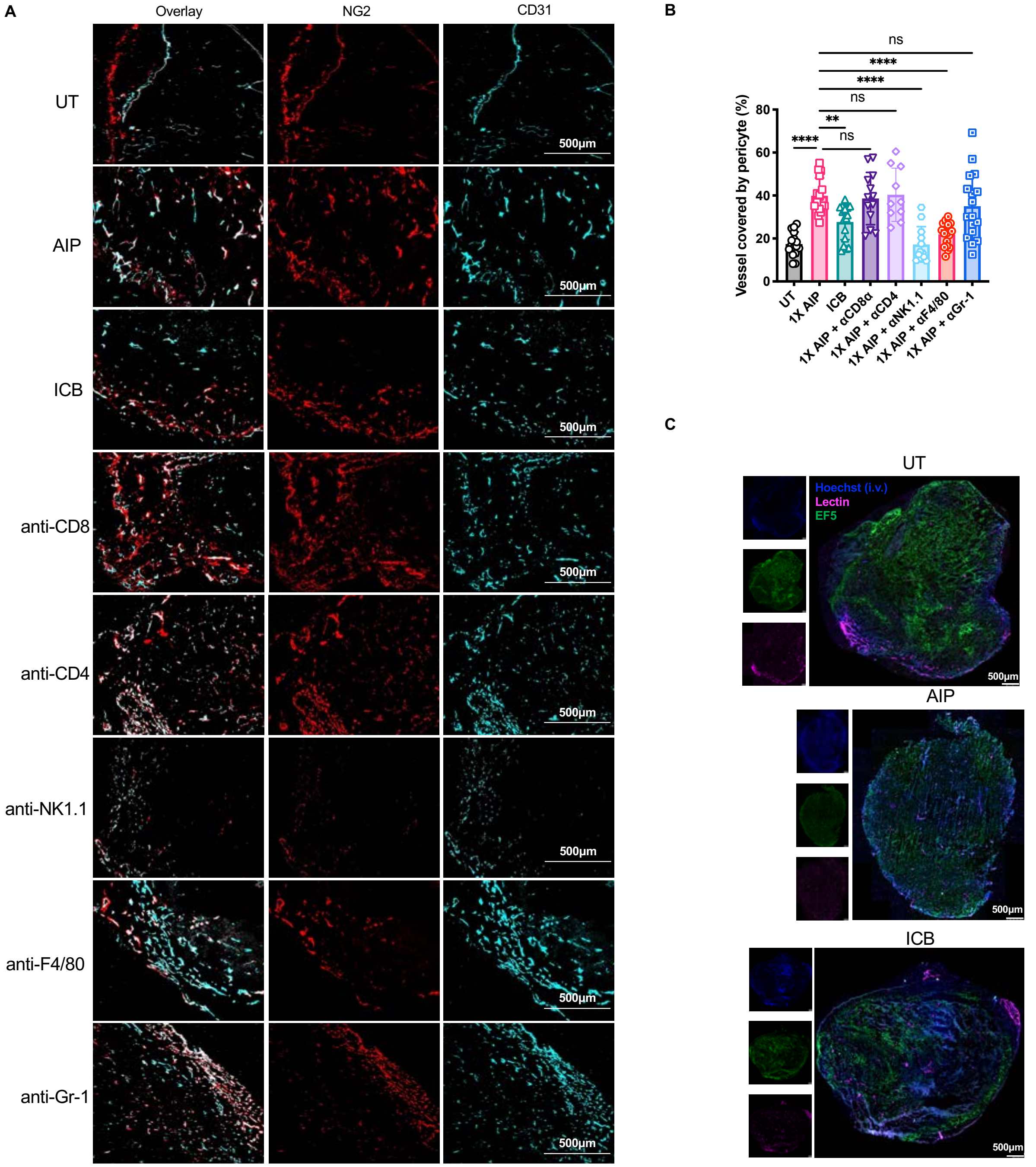

Figure S6, related to Figure 6. NK cells and macrophages contribute to vessel normalization following AIP treatment.

(**A**) Representative images of B16F10 tumors treated with AIP and indicated depletion antibodies (against $CD8^+$ T cells/$CD8\alpha^+$ DCs, CD4+ T cells, neutrophils, NK cells, and macrophages) for 3 days. Tumors were collected and cryo-sectioned into 8 μm thick slices and stained with DAPI, CD31 (cyan) and NG2 (red) antibodies.

(**B**) Fraction of vessels covered by pericytes are quantified by the colocalization level of CD31 and NG2 in tumor sections treated the same way as in (**A**). ***P*<0.01; *****P*<0.0001; by one-way ANOVA with the Dunnett test.

(**C**) B16F10-bearing mice were untreated or treated with AIP or ICB for 3 days before intravenously injected with a hypoxia-detecting agent EF5. Tumors were collected and cut into 8μm thick slices and stained with EF5 antibodies. AF647 labeled lectin was used to label vasculatures.

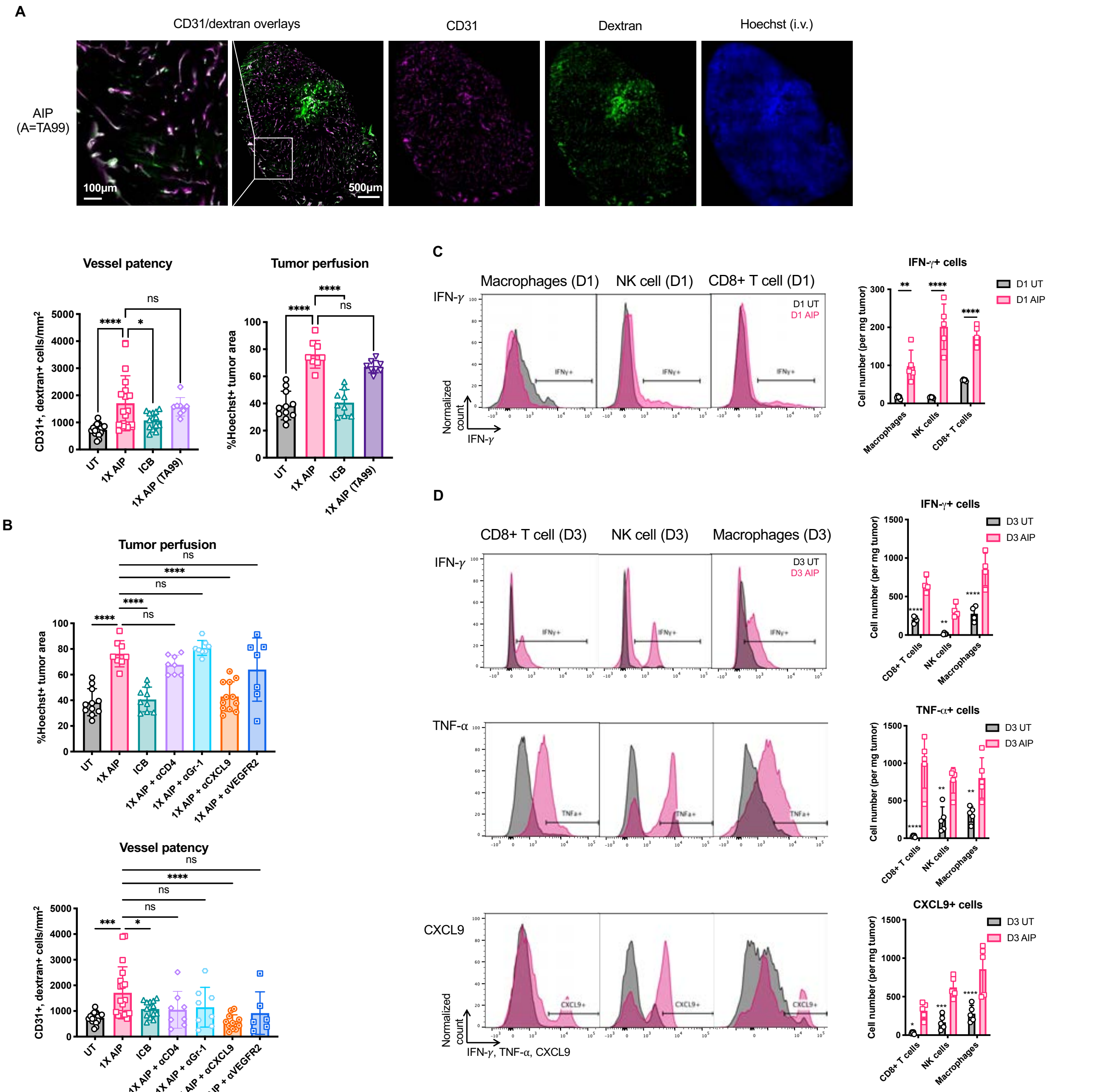

Figure S7, related to Figures 6 and 7. AIP therapy with TA99 antibody induces signs of vascular normalization similar to AIP treatment with 2.5F-Fc antibody surrogate.

(**A**) B16F10-bearing mice were injected with dextran and Hoechst before euthanasia as in Figure 6A and were treated with AIP, where A is a TYRP1-targeting TA99 antibody. Shown were quantifications of vessel patency (CD31$^+$, dextran$^+$ cells/mm^2, lower left) and tumor perfusion (Hoechst$^+$% area, lower right) levels in tumor sections.

(**B**) Mice with B16F10 tumors were treated with AIP and indicated depletion antibodies against CD8$^+$ T cells/CD8α$^+$ DCs, CD4$^+$ T cells, macrophages, NK cells, IFN-γ and TNF-α for 3 days before intravenously injected with 70kD dextran (red) and a DNA-staining dye Hoechst (blue) and perfused systematically with PBS. Tumors were collected and cut into 100 μm thick slices with a vibratome and stained with CD31 antibodies. Shown are quantification of tumor perfusion (left, Hoechst$^+$% area) and tumor vessel patency (right, CD31$^+$, dextran+ cells/mm^2) in tumor sections

from mice treated with ICB or AIP (with or without indicated depleting antibodies) as described above.
(**C-D**) B16F10-bearing mice were untreated or treated with single-dose AIP. Tumors were collected 1 day (**C**) or 3 days (**D**) post treatment. The number of IFN-γ-, TNF-α- and CXCL9-secreting macrophages, NK cells and CD8$^+$ T cells were determined by flow cytometry. **P*<0.05; ***P*<0.01; *****P*<0.0001; versus untreated by two-way ANOVA.
Throughout, **P*<0.05; ***P*<0.01; ****P*<0.001; *****P*<0.0001; versus 1X AIP by one-way ANOVA with the Dunnett post test (**A**-**B**) and versus untreated by two-way ANOVA with the Holm-Šídák test (**C-D**).
